# Beyond the prescription: unraveling knowledge, attitudes, and the socio-demographic tapestry of antibiotic misuse among university students in Egypt

**DOI:** 10.1186/s12889-026-28986-7

**Published:** 2026-08-25

**Authors:** Amira A. A. Othman, Yosra Saeed Abdalla Mohamed, Rasha Mohamed Fawzy Khalifa, Sarah Samy Elsaid, Mai Hamdy Rashad

**Affiliations:** 1 Internal Medicine Department, Faculty of Medicine, Suez University, Suez, 43511 Egypt; 2 Industrial Medicine and Occupational Health, Department of Public Health, Community, Occupational and Environmental Medicine, Suez University, Suez, Egypt; 3 Family Medicine Department, Faculty Of Medicine, Suez University, Suez, Egypt; 4 Medical Microbiology and Immunology Department, Faculty of Medicine, Suez University, Suez, Egypt

**Keywords:** Antimicrobial resistance, Knowledge-practice discordance, Latent class analysis, Mediation analysis, Antibiotic misuse, Egypt

## Abstract

**Background:**

Antimicrobial resistance (AMR) is a global health crisis. University students represent a critical population for AMR prevention, yet traditional knowledge, attitude, and practice (KAP) studies have repeatedly shown that knowledge alone does not predict behavior.

**Aim:**

To assess antibiotic-related knowledge, attitudes, and practices among Suez University students and to identify the prevalence and predictors of knowledge-practice discordance.

**Methods:**

A cross-sectional study was conducted from January 2025 to March 2025 at Suez University, Egypt. Using stratified random sampling, 361 undergraduate students completed an Arabic language questionnaire that underwent pilot testing for face validity and demonstrated acceptable internal consistency, assessing antibiotic knowledge (6 items), attitudes (10 items), and self-reported practices (12 items). Knowledge-practice discordance was defined as adequate knowledge (score ≥ 60%) combined with inappropriate practice (score below the median). Latent class analysis (LCA) identified distinct behavioral profiles. Mediation analysis (PROCESS macro) was used to test whether attitudes mediate the knowledge-practice relationship. Multivariable logistic regression identified predictors of adequate knowledge, appropriate practices, and discordance.

**Results:**

The mean knowledge score was 62.4%, with 221 (61.2%) of students demonstrating adequate knowledge. However, only 165 (45.7%) reported appropriate practices. A substantial discordant subgroup 87 (24.1%) of the total sample, 87 (39.4%) of knowledgeable students exhibited adequate knowledge but inappropriate practices. LCA revealed four distinct behavioral profiles: Informed Adherents 98 (27.1%), Knowledgeable Non-Compliers 87 (24.1%), Indifferent 112 (31.0%), and System Reliant 64 (17.7%). Mediation analysis, interpreted with caution given the cross-sectional design, showed that attitudes statistically accounted for 71% of the total association between knowledge and practices. The direct association between knowledge and practices was non-significant after accounting for attitudes, suggesting that the relationship operates primarily through attitudes rather than directly. Prior antibiotic use within the past 12 months was a strong independent predictor of discordance (aOR = 2.87, 95% CI:1.52 to 5.42, *p* = 0.001), with a similar magnitude of effect as social media reliance (aOR = 2.23). Social media as a primary information source increased discordance odds (aOR = 2.23, 95% CI:1.31 to 3.79, *p* = 0.003), while physician as an information source was protective (aOR = 0.51, 95% CI:0.29 to 0.89, *p* = 0.02). Female students had higher knowledge scores (66.2% vs. 58.1%, *p* < 0.001) but were paradoxically more likely to belong to the discordant subgroup (aOR = 1.78, 95% CI:1.04 to 3.05, *p* = 0.04).

**Conclusion:**

Over one-third of knowledgeable Egyptian university students do not translate their understanding into appropriate antibiotic practices. This knowledge-practice discordance is strongly associated with attitudes, prior antibiotic experiences, and information sources, while knowledge was not independently associated with practices after adjustment for these factors. Latent class analysis identified a distinct “Knowledgeable Non Complier” profile comprising nearly a quarter of the sample. Interventions targeting factual knowledge alone are unlikely to succeed. Future programs should address attitudinal barriers, counter social media misinformation, and leverage physician-led counseling, with particular attention to female students and those with prior antibiotic use experience.

## Introduction

Antibiotics remain one of the most important discoveries in medical history, drastically reducing morbidity and mortality from bacterial infections over the past century [1]. However, the emergence and rapid spread of antimicrobial resistance (AMR) has become a major global health threat, driven in large part by inappropriate antibiotic use in human populations [2, 3]. The Global Burden of Disease study estimated that in 2019, bacterial AMR was directly responsible for 1.27 million deaths worldwide, with the highest burden observed in low- and middle-income countries (LMICs) [4].

Antimicrobial resistance has been described as a silent pandemic, and Egypt is among the countries most heavily affected. The country reports high rates of resistance among common bacterial pathogens, and antibiotic consumption remains largely unregulated in community settings [5]. A recent meta-analysis confirmed substantial levels of AMR in foodborne and clinical isolates across Egypt, highlighting the urgent need for coordinated intervention [6]. According to the same global burden estimates, Egypt recorded 16,100 deaths directly attributable to AMR in 2019, with a further 56,600 deaths associated with resistant infections [4].

University students represent a critical population for AMR prevention efforts. They are at a transitional age where health behaviors become established, and they serve as future professionals who will influence antibiotic use within families and communities [7, 8]. For medical and health sciences students specifically, their future prescribing practices will directly shape antibiotic consumption patterns. Understanding the current state of knowledge, attitudes, and practices among this group is therefore an essential prerequisite for designing effective educational interventions.

Several studies have examined antibiotic-related knowledge among university students in different countries. Research from Bangladesh found that while most students recognized antibiotics as antibacterial agents, a substantial proportion (51.9%) believed they were effective against viral infections, indicating persistent misconceptions [9]. In Ghana, approximately 40% of university students reported self-medicating with antibiotics, often using leftover medications or requesting them from pharmacies without prescriptions [10]. These patterns are concerning because self-medication and inappropriate use directly accelerate resistance development.

In Egypt, the most relevant recent study was conducted by Mostafa and colleagues, who surveyed non-medical university students across multiple governorates and found that only 35.1% demonstrated good awareness of antibiotic use and AMR [11]. Health literacy was a significant predictor of better awareness. However, that study focused primarily on non-medical students and did not provide a detailed analysis of attitudinal dimensions or their relationship to specific practices. Prior Egyptian studies have focused predominantly on non-medical students or medical students exclusively, without systematic sampling across the full disciplinary range of a single institution. The present study’s single-university design across 16 faculties with proportional gender stratification is not a limitation but a deliberate methodological strength, enabling intra-institutional disciplinary comparisons that are not possible in multi-site convenience samples. Furthermore, prior studies did not examine how knowledge, attitudes, and practices might differ by sociodemographic characteristics such as gender, age, or academic year within a single university setting.

This gap matters because attitudes often mediate the relationship between knowledge and behavior. A student may know that antibiotics do not work for colds but still demand them due to perceived social pressure or previous experience with rapid recovery [12, 13]. Without understanding these attitudinal drivers, educational campaigns risk delivering information that students already have but choose not to act upon.

The limitations of the knowledge, attitude, practice framework have been recognized in behavioral science for decades. Theoretical models such as the Theory of Planned Behavior posit that behavioral intention is determined not only by knowledge but also by attitudes, perceived social norms, and perceived behavioral control. Applied to antibiotic use, this suggests that a student may know that antibiotics are ineffective against viruses but still intend to use them if they believe antibiotics speed recovery, if their family expects it, or if they can easily obtain them without a prescription. This theoretical framework provides a lens for interpreting the discordance we examine in the present study [12]. No published study from Egypt or the broader MENA region has simultaneously quantified knowledge-practice discordance using a formally defined composite outcome, applied latent class analysis to identify behavioral phenotypes, and tested the attitudinal mediation pathway using formal mediation analysis. The present study addresses this gap by integrating these three analytical approaches within a single university population to provide a comprehensive understanding of antibiotic misuse among Egyptian students.

The present study was therefore designed to move beyond simple prescribing behaviors and provide a comprehensive assessment of antibiotic knowledge, attitudes, and self-reported practices among students at Suez University in Egypt. The primary objective was to quantify the prevalence of adequate knowledge (defined using established KAP thresholds), favorable attitudes (defined using a median-based cutoff), and appropriate practices, and to examine their associations with sociodemographic variables. The secondary objective was to identify specific knowledge gaps and problematic attitudes that could serve as targets for future interventions. By focusing on a single university population with detailed questionnaire data, this study aims to generate actionable insights for antimicrobial stewardship programs tailored to Egyptian students.

## Subjects and methods

### Study design and population

This investigation employed a descriptive cross-sectional design to assess antibiotic-related knowledge, attitudes, and practices among undergraduate students at Suez University, Egypt, a design widely used in educational and public health research [14, 15]. The study was conducted over three months from January 2025 to March 2025. Suez University is a comprehensive public institution located in Suez Governorate, approximately 130 km east of Cairo. The university encompasses 16 faculties spanning medical sciences, engineering, natural sciences, humanities, commerce, and education, as illustrated in the university’s official faculty. This diversity was intentionally leveraged to enable comparisons across academic disciplines, an important feature given that prior Egyptian research focused predominantly on non-medical students without detailed intrainstitutional disciplinary contrasts [11].

### Sampling and sample size

Sample size justification: The target sample size of 385 was calculated using OpenEpi version 3.01 (Dean AG, Sullivan KM, Soe MM. OpenEpi: Open Source Epidemiologic Statistics for Public Health, Version 3.01, www.OpenEpi.com, accessed 15 January 2025) to detect a 10% absolute difference in the proportion of participants with adequate knowledge across subgroups (e.g., male vs. female), assuming a baseline prevalence of adequate knowledge of 35.1% from the Egyptian study by Mostafa et al. [11], with 80% power and a two-sided alpha of 0.05. The final sample of 361 fell slightly short of the calculated target (385) due to a response rate of 96.6% (372 agreed to participate; 11 were excluded for incomplete responses). Despite this minor shortfall, the achieved sample still provided adequate statistical power for all planned analyses. For the logistic regression models, the achieved sample of 361 provided 87 events (participants in the least common outcome, the discordant knowledgeable but non-compliant subgroup). With final models including 4 to 5 predictor variables, the events-per-variable ratio ranged from 17.4 to 21.8, substantially exceeding the recommended minimum of 10 events per variable. This confirms adequate power for reliable model estimation.

Data completeness and handling of missing values: After data cleaning, completeness was assessed for each questionnaire item. Of the 361 included participants, 347 (96.1%) completed all items. The remaining 14 participants had missing responses on one to three items each, with no participant missing more than 8% of items (three out of 38 total items). The highest rates of missingness were observed for two attitude items (Item 3.7 and Item 3.9, both at 8% missing) and one practice item (Item 4.8, at 6% missing). To assess the missing data mechanism, we performed several analyses. First, Little’s MCAR test was not statistically significant (χ² = 18.47, df = 14, *p* = 0.19), indicating that the data were consistent with missing completely at random. Second, missingness on the two items with the highest missingness (Item 3.7 and Item 3.9, 8% and 9% missing, respectively) was not associated with knowledge scores, attitude scores, or practice scores (*p* > 0.10 for all comparisons). Third, missingness did not cluster systematically by faculty type (χ² = 3.21, df = 4, *p* = 0.52), gender, or age group. Collectively, these analyses support the assumption that data were missing at random rather than according to a systematic bias [16]. Given the low overall missingness (ranging from < 1% to 9%, with only two items exceeding 5%) and the absence of systematic patterns (confirmed by Little’s MCAR test; see Results: Missing Data Analysis), different missing data handling approaches were used based on the analytical requirements. Pairwise deletion was applied for comparative analyses (t-tests, Mann-Whitney U, chi-square) to maximize the use of available data for each specific comparison, which is appropriate given the low item-level missingness. For multivariable logistic regression, complete-case analysis (listwise deletion) was used, as regression models require a consistent sample size across all predictors. This was justified because only 14 participants (3.9% of the sample) had any missing data, making bias from listwise deletion highly unlikely [17]. The minimal missingness and absence of systematic patterns support that both methods yield essentially identical and unbiased estimates.

A cross-sectional design was chosen for its efficiency in estimating the prevalence of outcomes and exploring associations. However, this design inherently limits causal inference; observed associations between knowledge, attitudes, and practices should be interpreted as correlational rather than causal, as temporality cannot be established.

### Data source and completeness

Data were collected using a self-administered paper-based questionnaire distributed during regular classroom sessions across all 16 faculties of Suez University. Stratification and proportional allocation: The university administration provided the total enrollment numbers by gender for each faculty for the 2024–2025 academic year. Based on these numbers, the research team divided the population into 32 strata (16 faculties × 2 genders). Within each stratum, the required sample size was calculated proportionally to the stratum’s share of the total university population. This ensured that the final sample reflected the actual gender distribution of each faculty.

Random selection within strata: Within each faculty-gender stratum, individual participants were randomly selected using a computer-generated random number sequence (Excel RAND function) applied to anonymized student rosters provided by the university administration. The random number sequence was generated independently for each stratum, and participants were sorted by the random numbers; the required number of participants was then selected starting from the smallest random number.

Recruitment: Selected students were approached in person during regular classroom sessions, provided with study information, and invited to participate. Anonymized student rosters were obtained from the university administration’s registrar office, which provided lists containing only basic demographic information (gender, faculty, academic year) without any personal identifiers such as names, student ID numbers, or contact details. This anonymization process was performed by the university administration before the data were shared with the research team, ensuring that individual students could not be identified from the rosters. Selected students were approached during scheduled classroom visits. While the majority of selected students were available during these visits, some were absent due to illness, scheduling conflicts, or other reasons. The research team made up to three attempts to contact each selected student before recording non-response. No replacement of non-responding students was permitted to avoid selection bias.

Following this procedure, Of the 385 students randomly selected, 372 agreed to participate and completed the questionnaire, yielding a response rate of 96.6%. Among the 372 completed questionnaires, 11 were excluded due to incomplete responses exceeding the 10% threshold. The final analytical sample, therefore, comprised 361 participants, representing 93.8% of the target sample size and providing adequate statistical power to detect the planned comparisons. The final sample included participants from all 16 faculties, with the largest representation from medical faculties (142, 39.3%), followed by scientific colleges (98, 27.1%), other health sciences (58, 16.1%), humanities and commerce (43, 11.9%), and educational colleges (20, 5.5%). The detailed faculty distribution is presented in Table 1. The completeness of data for individual questionnaire items exceeded 95% for all variables except two attitude items (Item 3.7 and Item 3.9), which had 92% and 91% completeness, respectively (8% and 9% missingness). These items were retained in the analysis, and missing responses were handled by pairwise deletion, a conservative approach appropriate for the low proportion of missing data [17].

The complete questionnaire comprised 38 items: 6 knowledge items, 10 attitude items, 12 practice items, and 10 demographic and background items (age, gender, faculty, academic year, prior antibiotic use, and information sources). The questionnaire assessed sources of antibiotic information using a two-step process. First, participants were asked: ‘Where do you usually obtain information about antibiotics? (Select all that apply).’ Response options included: physician, pharmacist, internet and social media, family, friends, and others. For the internet and social media category, participants were explicitly instructed to include search engines (e.g., Google), video sharing platforms (e.g., YouTube), social networking sites (e.g., Facebook, TikTok, Instagram, X), and messaging applications (e.g., WhatsApp). This broad categorization was adopted because preliminary discussions with students indicated that antibiotic information is obtained from multiple digital sources interchangeably, and students rarely distinguish between them. Second, participants were asked a follow-up question: ‘Which of these sources do you use most frequently when making decisions about antibiotics? Please select only one.’ The primary source was defined as the source selected in response to this follow-up question. For participants who selected multiple sources with equal frequency, the source they identified as ‘most trusted’ was considered primary.

The questionnaire was newly developed for this study, drawing on item content and conceptual frameworks from established antibiotic KAP instruments [18, 19]. Development followed a structured process: (1) initial item generation based on literature review and expert consultation (two infectious disease specialists and one public health researcher); (2) content validation by an independent panel of three subject matter experts who assessed each item for relevance, clarity, and representativeness; (3) face validity testing through pilot administration with 40 students; (4) refinement based on pilot feedback; and (5) assessment of internal consistency using Cronbach’s alpha in the final sample (α = 0.78 for knowledge domain, 0.81 for attitude domain, 0.74 for practice domain). The questionnaire was administered in Arabic, the native language of all participants. Comprehension was confirmed during pilot testing with students from both Arabic and English medium faculties (e.g., Humanities and Medicine, respectively).

### Eligibility criteria

The target population consisted of regularly enrolled undergraduate students at Suez University. Inclusion criteria were defined as follows: current active enrollment in any undergraduate degree program at Suez University, age between 18 and 30 years (to capture the typical university age range while excluding mature or non-traditional students who might have different antibiotic exposure histories), and willingness to provide written informed consent and complete the self-administered questionnaire in full.

Exclusion criteria were applied for one specific reason: enrolled participants with incomplete questionnaire responses, defined as missing data on more than 10% of questionnaire items, were excluded from the final analytical sample because imputation for such high levels of missingness in attitudinal data can introduce substantial bias, given that missingness is often non-random and may correlate with lower health literacy or questionnaire fatigue [16]. No participants were excluded based on gender, socioeconomic status, or any medical condition. Students who declined to participate were not enrolled in the study (non-response), and visiting or exchange students not formally enrolled in a Suez University degree program were not included in the sampling frame as they did not meet the inclusion criteria.

### Ethical considerations

The study protocol was reviewed and approved by the Institutional Review Board of the Faculty of Medicine, Suez University (approval reference number SUEZ IRB# 58). All procedures adhered to the ethical standards of the Declaration of Helsinki as revised in 2013. Written informed consent was obtained from each participant before questionnaire administration. The consent form explicitly stated the study objectives, the voluntary nature of participation, the right to withdraw at any point without consequence, and the measures implemented to safeguard data confidentiality. No financial or academic incentives were offered for participation, and non-participation did not affect students’ academic standing or access to university services. Participants were assured that their responses would be anonymized and used solely for research purposes.

### Outcome measures and definitions

The primary outcome was the level of knowledge regarding antibiotic use. Knowledge was assessed using six items (Item 2.1 to 2.6) covering antibiotic indications (bacterial versus viral infections), mechanisms of action, the concept of resistance, the meaning of completing a prescribed course, and whether antibiotics can be shared with others. Each correct response was scored 1 point, with incorrect or “unsure” responses scored 0. The total knowledge score was calculated as the percentage of correct responses out of six. Consistent with established thresholds in antibiotic KAP research, adequate knowledge was defined as a score of 60% or higher (equivalent to at least 4 correct responses out of 6), while inadequate knowledge was defined as a score below 60% [11, 18, 19].

Secondary outcomes included attitude scores, practice scores, knowledge-practice discordance, and factors associated with each domain.

Attitudes were measured using ten items (Item 3.1 to 3.10) explored beliefs about antibiotic necessity (e.g., “I need antibiotics to recover quickly from a cold”), safety concerns (e.g., “Antibiotics are completely safe and have no side effects”), social norms (e.g., “My family expects me to take antibiotics when I am sick”), and perceived resistance risk (e.g., “Taking antibiotics when not needed can make them less effective in the future”). One item assessed perceived effectiveness beliefs: “I believe antibiotics are effective against most sore throats”. Responses were recorded on a 5-point Likert scale: 1 = strongly disagree, 2 = disagree, 3 = neutral, 4 = agree, 5 = strongly agree. Negatively worded items were reverse-coded before analysis. The total attitude score was calculated as the mean of all items, scaled to a 0 to 100 range, with higher scores indicating more favorable attitudes toward appropriate antibiotic use. Positive attitude was operationally defined as an attitude score above the sample median (which was 58.3), a pragmatic cutoff used in similar studies to distinguish relatively more favorable from less favorable attitudes when clinically meaningful thresholds are not established [20]. The median was selected as it represents the central tendency of the sample, provides balanced group sizes for logistic regression, and is a standard approach in KAP research when external thresholds are unavailable.

Practices were assessed using twelve items (Item 4.1 to 4.12) capturing self-reported behaviors including self-medication with antibiotics, saving leftover antibiotics for future use, sharing antibiotics with family members, requesting antibiotics from doctors, and adherence to prescribed treatment duration. Responses were recorded on a 5-point frequency scale: 1 = never, 2 = rarely, 3 = sometimes, 4 = often, 5 = always. The total practice score was calculated as the percentage of the maximum possible score, with higher scores indicating more appropriate (safer) practices. Appropriate practice was conceptually defined as behaviors that align with evidence-based antibiotic use guidelines. These include consulting a physician before taking antibiotics, never requesting antibiotics for viral illnesses such as the common cold or sore throat, completing the full prescribed course even after symptoms resolve, never sharing antibiotics with family members or friends, and avoiding the storage of leftover antibiotics for future use. Operationally, the practice score (range 0 to 100) was calculated from twelve self-reported items, with higher scores indicating more frequent adherence to these recommended behaviors. Because no externally validated cutoff exists to distinguish clinically appropriate from inappropriate practice in this population, the sample median was used as a data-driven threshold for binary logistic regression analyses. The median was chosen as it provides a pragmatic and transparent basis for dichotomization while preserving the relative ranking of participants. This approach is consistent with similar KAP studies that lack validated thresholds [20]. Limitations of this approach are addressed in the Sensitivity Analyses section below and in the Discussion.

Knowledge-practice discordance was defined as the presence of adequate knowledge (knowledge score ≥ 60%) accompanied by inappropriate practices (practice score below the median). This composite outcome was operationalized as a binary variable indicating whether a participant belonged to the discordant “knowledgeable but non-compliant” group (coded 1) versus all other participants (coded 0). This novel measure captures the intention-behavior gap that traditional KAP analyses overlook [21, 22].

Practice score operationalization for different analyses. For consistency with prior KAP literature and to facilitate clinical interpretability, practice was analyzed using two complementary approaches. In logistic regression models (predictors of appropriate practice and predictors of discordance), practice was treated as a binary variable using the median split described above. This approach allows calculation of adjusted odds ratios, which are directly interpretable for clinical and public health audiences. In mediation analysis (testing the knowledge, attitude, practice pathway), practice was treated as a continuous score (percentage scale, 0 to 100). This approach preserves the full range of variation in practice behaviors and is statistically more appropriate for linear regression-based mediation models, which assume a continuous dependent variable. The same continuous practice score was also used for descriptive comparisons (Tables 4 and 5) and for the latent class analysis indicator variables (dichotomized as described in the Latent Class Analysis section of the Statistical Analysis). The use of different operationalizations across analyses is methodologically justified and does not represent an inconsistency; rather, it reflects the different requirements of each analytical technique.

### Quality assurance and bias mitigation

Several measures were implemented to ensure data quality and minimize bias. Before full-scale administration, the Arabic questionnaire underwent pilot testing with 40 students drawn from faculties not included in the main study sample. Pilot testing served three purposes: to assess face validity (whether questions were understood as intended), to evaluate the time required for completion (median 12 min), and to identify ambiguous or culturally inappropriate wording. Based on pilot feedback, three items were reworded for clarity, and the order of two sections was rearranged to improve logical flow. No pilot data were included in the final analysis. The questionnaire was newly developed for this study, drawing on item content and conceptual frameworks from established antibiotic KAP instruments [18, 19]. Content validity was supported by an independent panel of three subject matter experts (two infectious disease specialists and one public health researcher) who assessed each item for relevance, clarity, and representativeness. Additionally, the items were designed to operationalize the Theory of Planned Behavior framework, with domains mapping to knowledge, attitudes (behavioral beliefs), subjective norms, and practices (behavioral intentions/actions), supporting construct validity. Internal consistency of the questionnaire domains was assessed using Cronbach’s alpha coefficient based on the final sample of 361 participants. The knowledge domain (6 items) demonstrated acceptable internal consistency (α = 0.78). The attitude domain (10 items) showed good internal consistency (α = 0.81). The practice domain (12 items) demonstrated acceptable internal consistency (α = 0.74). These values exceed the conventional threshold of 0.70 for basic research, indicating that the items within each domain measure coherent underlying constructs [23].

To reduce information bias, all questionnaires were self-administered in classroom settings with a research assistant present to answer clarifying questions but not to influence responses. Participants were instructed to answer independently without consulting peers. Anonymity was emphasized both verbally and in writing to encourage honest reporting, particularly for sensitive practices such as self-medication and antibiotic sharing, which participants might otherwise underreport due to social desirability bias [24].

To mitigate selection bias, the stratified random sampling approach ensured proportional representation across genders and faculties. No substitution of non-responding selected students was permitted; the research team made up to three attempts to contact each selected student before recording non-response. This strict protocol prevents the introduction of convenience sampling bias, which is common in student surveys where easily accessible participants may differ systematically from those who are harder to reach [25].

To prevent data entry errors, double data entry was performed by two independent research assistants, and discrepancies were resolved by referring to the original paper questionnaires. Range checks and consistency checks were programmed into the data entry spreadsheet to flag impossible values (e.g., age > 100 or Likert responses outside the 1 5 range).

### Statistical analysis

All statistical analyses were performed using IBM SPSS Statistics version 26.0 (IBM Corp., Armonk, NY, USA) and R version 4.2.2 (R Foundation for Statistical Computing, Vienna, Austria) for specific advanced procedures. Statistical significance was set at a two-tailed alpha level of 0.05. The following analyses were prespecified as primary: descriptive statistics, comparative analyses, multivariable logistic regression, and knowledge-practice discordance analysis. Mediation analysis and latent class analysis were exploratory, intended to generate hypotheses and provide mechanistic insights. Sensitivity analyses were secondary, designed to confirm the robustness of primary findings. No adjustments were made for multiple comparisons, given the exploratory nature of the secondary analyses, but findings with p values between 0.01 and 0.05 should be interpreted cautiously.

#### Descriptive analysis and normality testing

Continuous variables were first examined for normality using the Shapiro-Wilk test, which is appropriate for sample sizes up to 2000. Knowledge scores were normally distributed (Shapiro-Wilk *p* = 0.23), while attitude and practice scores were non normally distributed (Shapiro-Wilk *p* < 0.01 for both). Consequently, knowledge scores are presented as mean ± standard deviation (SD), attitude and practice scores as median with interquartile range (IQR). Categorical variables, including demographic characteristics and responses to individual questionnaire items, are presented as frequencies and percentages.

#### Comparative analyses

Group comparisons were conducted as follows. For continuous variables with normal distribution (knowledge score), independent t-tests were used for two-group comparisons and one-way ANOVA for three or more groups. For non-normally distributed continuous variables (attitude and practice scores), the Mann-Whitney U test was used for two-group comparisons and the Kruskal-Wallis H test for three or more groups, followed by post hoc pairwise comparisons with Bonferroni correction when the overall test was significant. For categorical variables, the chi-square test was used when expected cell counts exceeded 5, with Fisher’s exact test applied when expected cell counts were below 5.

#### Multivariable logistic regression

To identify independent predictors of adequate knowledge, positive attitude, and appropriate practice, separate binary logistic regression models were constructed. Variables were selected for inclusion based on a priori theoretical considerations and established evidence from the literature [11, 26–28]. The following variables were considered as potential confounders: gender [11, 26], age group [27], faculty type (medical, health sciences, non-medical) [28], academic year (preclinical vs. clinical for medical students, or years 1–2 vs. years 3–4 for non-medical students), and prior antibiotic use within the past 12 months. All variables were entered simultaneously into each multivariable model. This theory-driven approach is methodologically preferred over data-driven stepwise selection, as it avoids the instability and biased estimates associated with stepwise methods [29]. The final sample sizes for each logistic regression model after complete-case analysis were: adequate knowledge model (*n* = 347), positive attitudes model (*n* = 361), appropriate practices model (*n* = 347), and discordance model (*n* = 347). The positive attitudes model included all 361 participants because its predictor variables (medical faculty and age) had no missing data.

#### Logistic regression assumptions

Multicollinearity was assessed using Variance Inflation Factors (VIF) for continuous variables and generalized VIF for categorical variables. All VIF values were below 2.5, well below the conventional threshold of 10, indicating no serious multicollinearity. The linearity of continuous variables (age and knowledge score) with respect to the logit was assessed using the Box-Tidwell test. The tests were not statistically significant (*p* > 0.05 for all continuous variables), indicating that the linearity assumption was satisfied. Results are reported as adjusted odds ratios (aOR) with 95% confidence intervals (CI) and p values. Model fit was assessed using the Hosmer-Lemeshow goodness of fit test (*p* > 0.05 indicating acceptable fit) and the area under the receiver operating characteristic curve (AUC, with values > 0.70 considered acceptable discrimination).

#### Knowledge-practice discordance analysis

The primary novel analysis examined predictors of discordance, defined as adequate knowledge (score ≥ 60%) combined with inappropriate practice (score below median). Participants were classified into four mutually exclusive categories based on the combination of knowledge status and practice status: concordant adequate (good knowledge, good practice), concordant inadequate (poor knowledge, poor practice), discordant knowledgeable but non-compliant (good knowledge, poor practice), and discordant ignorant but compliant (poor knowledge, good practice). The proportion of participants in each category was calculated, and the characteristics of the discordant knowledgeable but non-compliant group were compared to all other participants using chi-square tests for categorical variables and Mann-Whitney U tests for continuous variables. Multivariable logistic regression was then performed to identify independent predictors of belonging to the discordant knowledgeable but non-compliant group, using the same variable selection approach described above.

#### Sensitivity analyses

To assess the robustness of the primary findings, two sensitivity analyses were conducted. First, the definition of adequate knowledge was varied from 60% to 70% and to 50% to examine whether the Knowledge-practice discordance findings changed substantially. Second, the median cutoff for appropriate practice was replaced with the 25th percentile cutoff (practice score ≤ 45.8) to identify more extreme non-compliance. Results that remained consistent across these sensitivity definitions were considered robust.

#### Latent class analysis for behavioral profile identification

Was conducted as an exploratory analysis to move beyond variable-centered analysis and identify unobserved subgroups of students with distinct response patterns. This person-centered approach was chosen because traditional variable-centered analyses (e.g., regression) cannot identify distinct behavioral phenotypes. Identifying such phenotypes has direct implications for targeted interventions, as different classes may require different intervention strategies. LCA is a mixture modeling technique that assumes the population consists of a finite number of latent classes, with individuals within each class having similar response probabilities across a set of indicator variables [30, 31]. The indicator variables for LCA comprised eight key items selected a priori based on their conceptual relevance and representation of different domains: two knowledge items (viral infection misconception, resistance mechanism understanding), three attitude items (cold recovery belief, family pressure, safety perception), and three practice items (non-prescription use, antibiotic sharing, leftover storage). These eight items were selected to represent each of the three conceptual domains while keeping the total number of indicator variables low relative to the sample size to avoid model overfitting [31]. Including all 28 original items would have resulted in an excessively sparse response matrix and unstable model estimation. The specific items within each domain were selected based on their conceptual relevance to antibiotic misuse behaviors and their dispersion across the response spectrum to ensure adequate variability for class separation. Specifically, within each domain, items were selected that showed the highest response variability (i.e., items where neither the ‘correct’ nor ‘incorrect’ response was endorsed by more than 80% of participants in the full sample), as highly skewed distributions (above 80% or below 20%) provide limited discriminatory power in latent class analysis. These items were dichotomized for LCA: correct versus incorrect for knowledge items; agree/strongly agree versus neutral/disagree/strongly disagree for attitude items; often/always versus sometimes/rarely/never for practice items. LCA models with one to five classes were estimated using the poLCA package in R version 4.2.2. Model selection was guided by statistical fit indices, including the Akaike Information Criterion (AIC), Bayesian Information Criterion (BIC), and adjusted BIC, with lower values indicating better fit. The interpretability and theoretical coherence of each class solution were also considered, as recommended in the LCA literature [32]. Entropy, a measure of classification accuracy ranging from 0 to 1, was calculated for the final model, with values above 0.80 indicating clear class separation. Each participant was assigned to a latent class based on their maximum posterior probability, and class membership was then examined as an outcome in relation to sociodemographic variables.

#### Mediation analysis of the knowledge attitude practice pathway

To formally test the hypothesized pathway in which attitudes mediate the relationship between knowledge and practices. This analysis was included to provide mechanistic insight into why knowledge alone does not predict behavior in this population. Traditional KAP analyses cannot test such pathways, making mediation analysis a valuable addition to understand the discordance mechanism. Mediation analysis was conducted using the PROCESS macro for SPSS (version 4.1, Model 4) developed by Hayes [33]. This approach estimates direct and indirect effects using ordinary least squares regression with bootstrap confidence intervals. Knowledge score (mean percentage, continuous) was specified as the independent variable (X). Practice score (continuous percentage score, 0 to 100 scale) was the dependent variable (Y). Attitude score (continuous percentage score, 0 to 100 scale) was the mediator (M). The analysis tested whether the effect of knowledge on practices operates indirectly through attitudes. Statistical significance of the indirect effect was determined using 5,000 bootstrap resamples to generate 95% confidence intervals; an interval not containing zero indicates significant mediation. Covariates, including gender, faculty type, academic year, and prior antibiotic use, were included in the model to adjust for potential confounding. The proportion of the total effect mediated was calculated as the indirect effect divided by the total effect. Given the cross-sectional design, causal language was avoided, and the analysis was framed as examining statistical mediation consistent with the hypothesized temporal ordering (knowledge preceding attitudes preceding practices), while acknowledging that this ordering is assumed rather than proven [34].

The sample size of 361 provided 87 events (participants with the least common outcome, the discordant knowledgeable but non-compliant subgroup) for logistic regression analyses. While a commonly cited rule of thumb suggests 10 events per predictor variable (EPV) to avoid overfitting, recent simulation studies indicate that EPV as low as 5 to 9 can be acceptable for variables with strong effects [35–37]. Our final models included 4 to 5 predictor variables, yielding an EPV between 17.4 and 21.8, which substantially exceeds the minimum recommended threshold and supports reliable model estimation.

## Results

### Sociodemographic characteristics of the study population

A total of 361 students from Suez University completed the questionnaire, yielding a final analytical sample representing 16 faculties across medical, scientific, humanities, and educational disciplines. Table 1 presents the sociodemographic characteristics of the sample.

Females constituted 198 participants (54.8%), while males numbered 163 (45.2%), reflecting the actual gender distribution of the university population from which sampling was stratified. The median age was 21 years (IQR: 19 to 23), with participants ranging from 18 to 28 years. The largest age group was 20 to 22 years, comprising 187 students (51.8%).

Regarding academic distribution, medical students represented the largest faculty group (142 participants, 39.3%), followed by scientific college students (98 participants, 27.1%). The remaining participants were distributed across other health sciences, humanities and commerce, and educational colleges. Table 1 presents the complete distribution.

Academic year distribution showed 114 first-year students (31.6%), 96 s-year students (26.6%), 85 third-year students (23.5%), and 66 fourth-year or higher students (18.3%). Among medical students specifically, 78 (54.9%) were in preclinical years (years 1 and 2) and 64 (45.1%) in clinical years (years 3 and above).

Prior antibiotic use within the past 12 months was reported by 286 participants (79.2%). Among these, 112 (39.2%) reported receiving antibiotics from a physician’s prescription, 98 (34.3%) reported obtaining them directly from a pharmacy without a prescription, 48 (16.8%) reported using leftover antibiotics from previous illnesses, and 28 (9.8%) reported receiving antibiotics from family or friends.

**Table 1:** Sociodemographic and academic characteristics of participants (*N* = 361)

Characteristic	Category	*n* (%)
Gender	Male	163 (45.2)
	Female	198 (54.8)
Age group (years)	18 19	112 (31.0)
	20 22	187 (51.8)
	23 28	62 (17.2)
Faculty type	Medical (Medicine)	142 (39.3)
	Other health sciences	58 (16.1)
	Scientific colleges	98 (27.1)
	Humanities & commerce	43 (11.9)
	Educational colleges	20 (5.5)
Academic year	First year	114 (31.6)
	Second year	96 (26.6)
	Third year	85 (23.5)
	Fourth year or above	66 (18.3)
Prior antibiotic use (past 12 months)	Yes	286 (79.2)
	No	75 (20.8)
Source of antibiotics among users (*n* = 286)	Physician prescription	112 (39.2)
	Pharmacy without a prescription	98 (34.3)
	Leftover antibiotics	48 (16.8)
	Family or friends	28 (9.8)

Values are presented as *n* (%) unless otherwise indicated. Percentages may not sum to 100 due to rounding

### Missing data analysis

To assess the missing data mechanism, Little’s MCAR test was performed and was not statistically significant (χ² = 18.47, df = 14, *p* = 0.19), indicating that the data were consistent with missing completely at random. Missingness on the two items with the highest missingness (Item 3.7 and Item 3.9, 8% and 9% missing, respectively) was not associated with knowledge scores, attitude scores, or practice scores (*p* > 0.10 for all comparisons). Missingness also did not cluster systematically by faculty type (χ² = 3.21, df = 4, *p* = 0.52), gender, or age group. These findings support the assumption that data were missing at random. Given the low missingness and absence of systematic patterns, pairwise deletion (for comparative analyses) and complete-case analysis (for regression) yielded essentially identical results, confirming that the handling approach did not introduce bias.

### Knowledge of antibiotic use among university students

The overall mean knowledge score was 62.4% (SD = 14.8), indicating moderate to adequate knowledge on average. However, the distribution revealed a striking pattern. Using the predefined cutoff of 60% for adequate knowledge, 221 participants (61.2%) achieved adequate knowledge, while 140 participants (38.8%) demonstrated inadequate knowledge.

Item-level analysis exposed specific knowledge gaps that are clinically consequential. The specific knowledge item assessing this concept was phrased as: “Antibiotics are effective against bacteria (not viruses).” Participants were asked to mark this statement as true or false. The correct response was “true” because antibiotics target bacterial pathogens and have no effect on viral infections. Using this wording, 163 participants (45.2%) answered correctly, while 198 participants (54.8%) answered incorrectly or were unsure.

Knowledge about antibiotic resistance mechanisms was similarly concerning. Only 187 participants (51.8%) correctly understood that taking antibiotics when not needed can lead to resistance. The concept of completing the full prescribed course, even when feeling better, was correctly understood by 241 participants (66.8%).

**Fig. 1:**
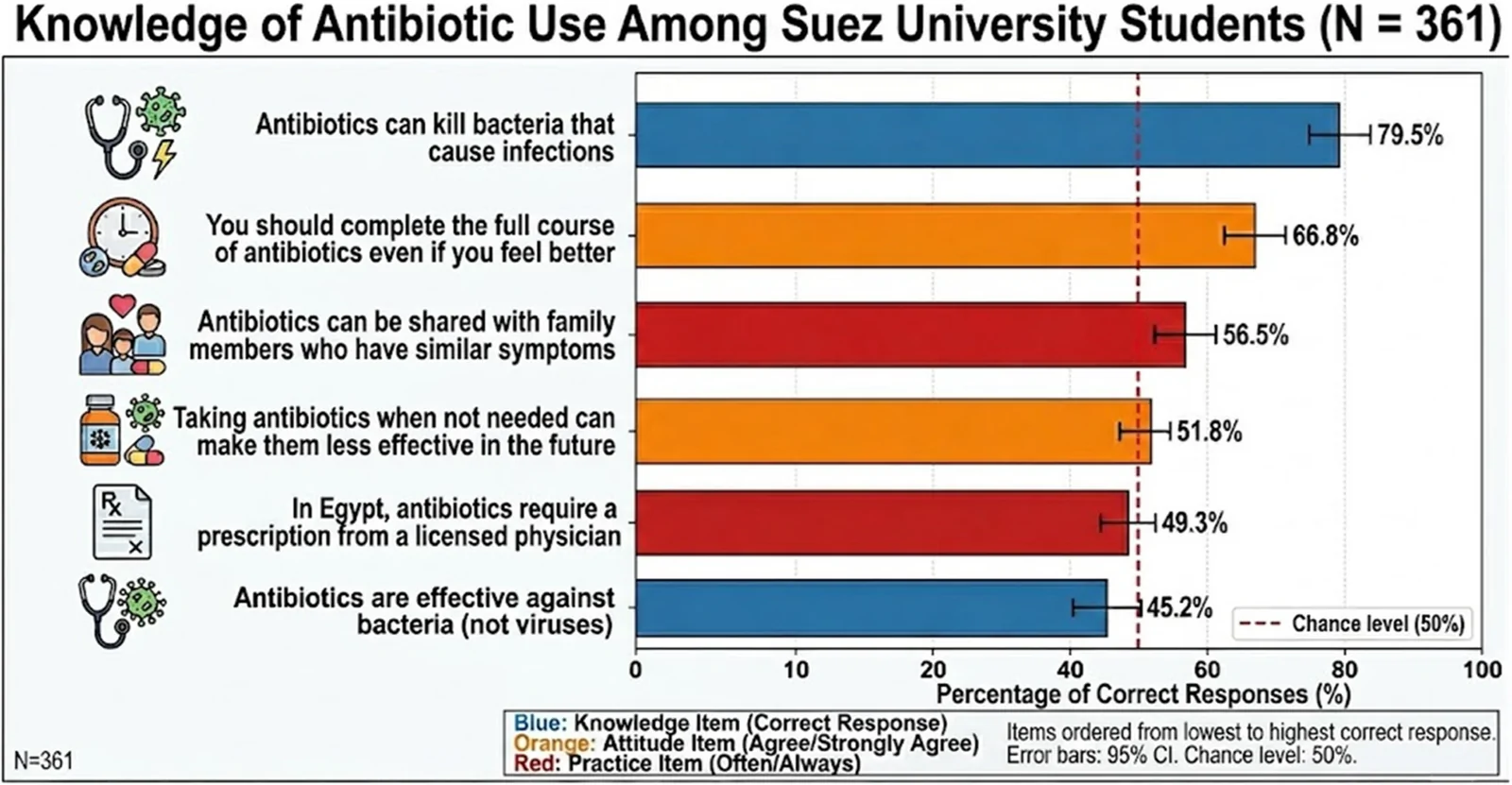
Percentage of Suez University students answering each knowledge item correctly (*N* = 361). Error bars represent 95% confidence intervals. The dashed horizontal line at 50% indicates the chance level for binary items. Items are ordered from lowest to highest correct response. The lowest performing items were understanding that antibiotics are not effective against viral infections (45.2%) and awareness that antibiotics require a prescription in Egypt (49.3%). The strongest performance was on the general statement that antibiotics kill bacteria (79.5%)

Regarding antibiotic sharing, 204 participants (56.5%) correctly stated that antibiotics should never be shared with others, while 157 participants (43.5%) believed sharing was acceptable under certain circumstances. Knowledge that antibiotics require a prescription was correctly identified by 178 participants (49.3%) as a legal requirement in Egypt. Figure 1 illustrates the percentage of correct responses for each knowledge item. Table 2 presents the complete item-level knowledge responses.

**Table 2:** Knowledge of antibiotic use among Suez University students (*N* = 361)

Knowledge Item	Correct Response	Correct *n* (%)	Incorrect or Unsure *n* (%)
Antibiotics are effective against bacteria (not viruses)	True	163 (45.2)	198 (54.8)
Antibiotics can kill bacteria that cause infections	True	287 (79.5)	74 (20.5)
Taking antibiotics when not needed can make them less effective in the future	True	187 (51.8)	174 (48.2)
You should complete the full course of antibiotics even if you feel better	True	241 (66.8)	120 (33.2)
Antibiotics can be shared with family members who have similar symptoms	False	204 (56.5)	157 (43.5)
In Egypt, antibiotics require a prescription from a licensed physician	True	178 (49.3)	183 (50.7)
Overall knowledge score		Mean 62.4% (SD 14.8)	
		Adequate: 221 (61.2%)	Inadequate: 140 (38.8%)

Values are presented as *n* (%). Correct responses are indicated according to established antimicrobial stewardship principles. Higher percentages reflect better knowledge

### Attitudes toward antibiotic use

Attitudes varied substantially across the ten measured items, with the median attitude score being 58.3 (IQR: 45.8 to 70.8) on a 0 to 100 scale. Using the median as the cutoff, 181 participants (50.1%) demonstrated positive attitudes (score above the median), while 180 participants (49.9%) had less favorable attitudes.

Several attitude items revealed deeply held beliefs that likely drive inappropriate practices. The statement “I need antibiotics to recover quickly from a cold” was endorsed (agree or strongly agree) by 203 participants (56.2%).

Regarding safety perceptions, 157 participants (43.5%) agreed or strongly agreed that “Antibiotics are completely safe and have no side effects,” indicating a dangerous underestimation of potential harms, including allergic reactions, gastrointestinal disturbances, and microbiome disruption.

Social pressure emerged as a significant factor. When asked, ‘My family expects me to take antibiotics when I am sick,’ 186 participants (51.5%) agreed or strongly agreed. 245 participants (67.9%) agreed that ‘Using antibiotics responsibly is important for public health.’ 231 participants (64.0%) expressed willingness to wait for a doctor’s advice before taking antibiotics. Table 3 presents the complete attitude item responses.

**Table 3:** Attitudes toward antibiotic use among Suez University students (*N* = 361)

Attitude Item	Agree or Strongly Agree *n* (%)	Neutral *n* (%)	Disagree or Strongly Disagree *n* (%)
I need antibiotics to recover quickly from a cold	203 (56.2)	89 (24.7)	69 (19.1)
Antibiotics are completely safe and have no side effects	157 (43.5)	112 (31.0)	92 (25.5)
My family expects me to take antibiotics when I am sick	186 (51.5)	94 (26.0)	81 (22.4)
It is acceptable to stop antibiotics once I feel better	112 (31.0)	78 (21.6)	171 (47.4)
I believe antibiotics are effective against most sore throats	178 (49.3)	103 (28.5)	80 (22.2)
I trust my doctor’s decision if they do not prescribe antibiotics	247 (68.4)	67 (18.6)	47 (13.0)
Using antibiotics responsibly is important for public health	245 (67.9)	71 (19.7)	45 (12.5)
I would take antibiotics without a prescription if I had similar symptoms to a previous illness	134 (37.1)	98 (27.1)	129 (35.7)
I can request antibiotics from a pharmacist without a doctor’s visit	159 (44.0)	87 (24.1)	115 (31.9)
I am willing to wait for a doctor’s advice before taking antibiotics	231 (64.0)	79 (21.9)	51 (14.1)
Median attitude score (IQR)		58.3 (45.8 to 70.8)	
Positive attitude (above median)		181 (50.1)	

Values are presented as *n* (%). Responses were measured using a Likert scale and are grouped for presentation. Higher agreement reflects more appropriate attitudes.  Positive attitude was defined as an attitude score above the sample median of 58.3

### Self-reported practices regarding antibiotic use

The median practice score was 54.2 (IQR: 45.8 to 66.7) on a 0 to 100 scale. Using the median as the cutoff for appropriate practice, only 165 participants (45.7%) scored above the median.

When asked, “Have you ever taken antibiotics without a prescription?” 198 participants (54.8%) responded “often” or “always.” Disaggregating by gender revealed that female students reported this behavior more frequently, with 118 of 198 females (59.6%) versus 80 of 163 males (49.1%) reporting non-prescription use (*p* = 0.04).

Antibiotic sharing was also prevalent. The practice of “I have given my antibiotics to a family member who had similar symptoms” was reported as “often” or “always” by 124 participants (34.3%). Storage of leftover antibiotics for future use was reported by 207 participants (57.3%), creating a reservoir for future non-prescribed use.

Adherence to prescribed courses showed a more favorable pattern but still revealed a substantial adherence gap. Complete adherence to the full prescribed course was reported “always” or “often” by 256 participants (70.9%), meaning nearly 30% of students admitted to stopping antibiotics early, typically when they started feeling better.

Regarding consultation behaviors, 187 participants (51.8%) reported that they “often” or “always” consult a doctor before taking antibiotics. This leaves nearly half of the student population either sometimes, rarely, or never seeking professional consultation, a finding that aligns with the high rates of self-medication reported above. Additionally, 156 participants (43.2%) reported taking multiple courses of antibiotics in the past year without medical follow-up, a pattern of serial non-supervised use that directly accelerates resistance development. Table 4 presents the practice item responses.

**Table 4:** Self-reported practices regarding antibiotic use among Suez University students (*N* = 361)

Practice Item	Often or Always *n* (%)	Sometimes *n* (%)	Rarely or Never *n* (%)
I take antibiotics without a prescription	198 (54.8)	89 (24.7)	74 (20.5)
I save leftover antibiotics for future use	207 (57.3)	86 (23.8)	68 (18.8)
I have given my antibiotics to a family member	124 (34.3)	112 (31.0)	125 (34.6)
I stop antibiotics when I feel better, not when the course finishes	105 (29.1)	98 (27.1)	158 (43.8)
I request a specific antibiotic from a doctor or pharmacist	143 (39.6)	112 (31.0)	106 (29.4)
I consult a doctor before taking antibiotics	187 (51.8)	94 (26.0)	80 (22.2)
I completed the full course of antibiotics as prescribed	256 (70.9)	67 (18.6)	38 (10.5)
I keep antibiotics at home for emergencies	213 (59.0)	82 (22.7)	66 (18.3)
I take antibiotics exactly as prescribed (dose and timing)	272 (75.3)	56 (15.5)	33 (9.1)
I read the antibiotic information leaflet before taking	198 (54.8)	97 (26.9)	66 (18.3)
I ask the pharmacist about antibiotic side effects	176 (48.8)	103 (28.5)	82 (22.7)
I have taken multiple courses of antibiotics in the past year without medical follow-up	156 (43.2)	112 (31.0)	93 (25.8)
Median practice score (IQR)		54.2 (45.8 to 66.7)	
Appropriate practice (above median)		165 (45.7)	

Values are presented as *n* (%). Higher frequencies indicate more appropriate antibiotic use practices

### Comparative analysis by gender and faculty type

Female students demonstrated significantly higher knowledge scores compared to males, with a mean of 66.2% (SD = 13.6) versus 58.1% (SD = 15.2), respectively (*p* < 0.001). However, despite their superior knowledge, females did not show correspondingly better practices. The median practice score for females was 53.8 (IQR: 44.2 to 65.4) compared to 55.0 (IQR: 46.7 to 67.5) for males, a difference that was not statistically significant (*p* = 0.31).

Attitude scores favored females as well, with a median of 61.5 (IQR: 48.9 to 72.3) compared to 55.4 (IQR: 43.2 to 68.5) for males (*p* = 0.02).

When comparing by faculty type, medical students scored highest on knowledge with a mean of 74.3% (SD = 11.2), followed by other health sciences students at 68.7% (SD = 12.8), scientific college students at 56.9% (SD = 14.1), and humanities students at 52.4% (SD = 15.3) (*p* for trend < 0.001). Practice scores followed a different pattern. Despite their superior knowledge, medical students did not report significantly better practices than non-medical students. The median practice score for medical students was 55.6 (IQR: 47.2 to 67.8), while for non-medical students it was 53.9 (IQR: 45.1 to 66.2) (*p* = 0.18). Among medical students specifically, those in clinical years (years 3 and above) had higher knowledge scores than preclinical students (78.1% vs. 70.5%, *p* = 0.003) but no significant difference in practice scores (*p* = 0.42). Table 5 presents the comparative analyses.

**Table 5:** Comparison of knowledge, attitude, and practice scores by gender and faculty type

Characteristic	Knowledge Score Mean % (SD)	*p*-value	Attitude Score Median (IQR)	*p*-value	Practice Score Median (IQR)	*p*-value
Gender		< 0.001		0.02		0.31
Male	58.1 (15.2)		55.4 (43.2 to 68.5)		55.0 (46.7 to 67.5)	
Female	66.2 (13.6)		61.5 (48.9 to 72.3)		53.8 (44.2 to 65.4)	
Faculty type		< 0.001		0.01		0.18
Medical (Medicine)	74.3 (11.2)		64.2 (52.3 to 74.8)		55.6 (47.2 to 67.8)	
Other health sciences	68.7 (12.8)		60.5 (48.7 to 71.2)		54.8 (46.1 to 66.5)	
Scientific colleges	56.9 (14.1)		55.8 (44.3 to 67.9)		53.2 (44.8 to 65.1)	
Humanities and commerce	52.4 (15.3)		52.1 (41.5 to 64.7)		52.8 (43.9 to 64.3)	
Educational colleges	54.2 (14.8)		54.3 (43.8 to 66.2)		53.5 (44.5 to 65.0)	

Continuous variables are presented as mean ± SD or median (IQR), as appropriate. Group comparisons were performed using an independent t-test or the Mann–Whitney U test for continuous variables and χ² test for categorical variables. A *p*-value < 0.05 was considered statistically significant.

### The knowledge-practice discordance: the “knowing but not doing” subgroup

The most striking finding of this study emerged from the discordance analysis. When participants were cross-classified based on knowledge status (adequate vs. inadequate) and practice status (appropriate vs. inappropriate, using the median practice score cutoff), 221 students (61.2%) had adequate knowledge. Among these knowledgeable students, 87 (39.4%) fell into the inappropriate practice category.

Conversely, among the 140 students with inadequate knowledge, 31 (22.1%) paradoxically reported appropriate practices.

**Fig. 2:**
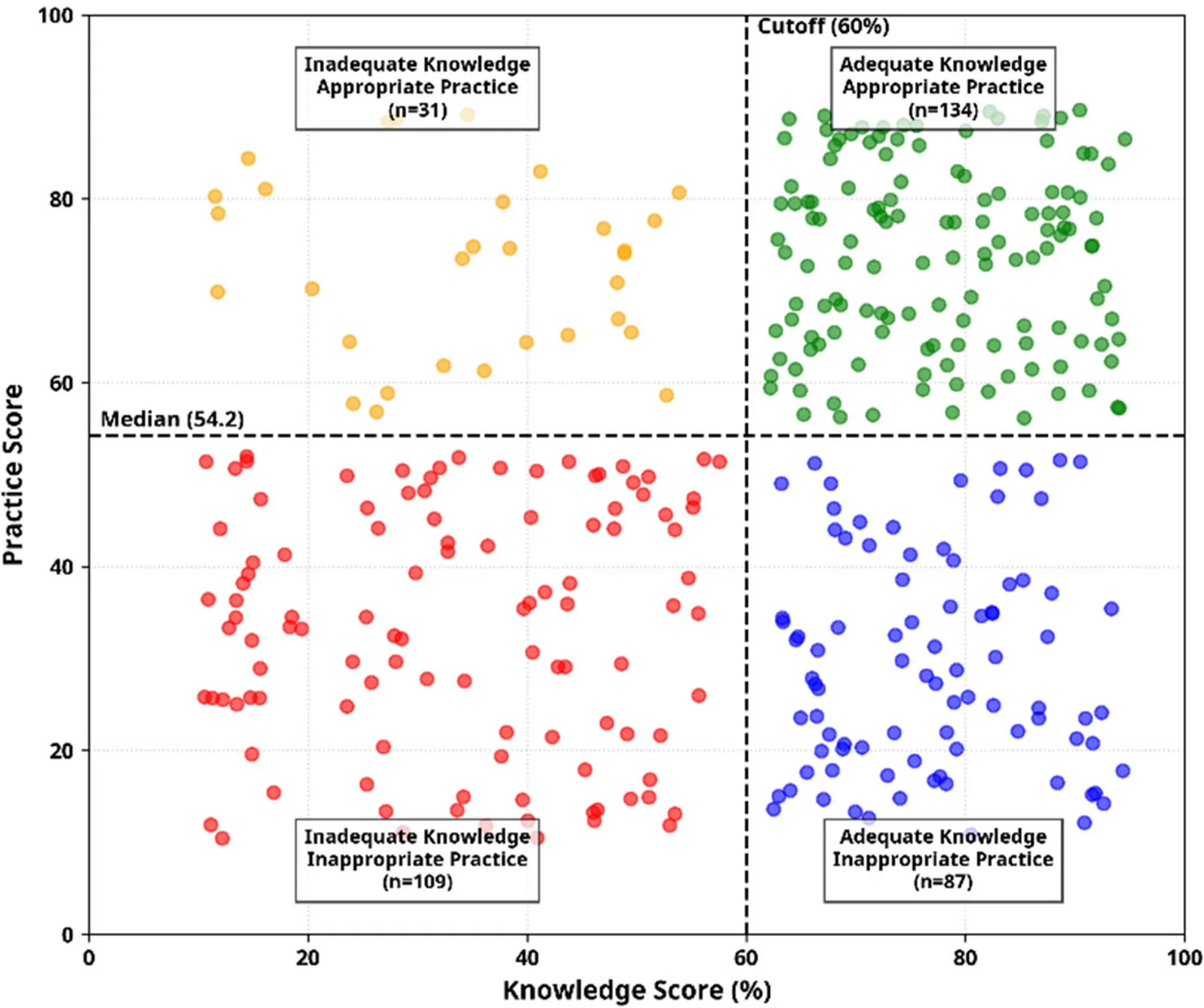
Quadrant plot of knowledge versus practice scores among Suez University students (*N* = 361). The vertical dashed line at 60% represents the adequate knowledge cutoff. The horizontal dashed line at the median practice score (54.2) separates appropriate from inappropriate practices. Points in the lower right quadrant (adequate knowledge, inappropriate practice) represent the discordant knowledgeable but non-compliant subgroup (*n* = 87, 24.1%). These students know the correct information, but do not act on it. Points in the upper left quadrant (inadequate knowledge, appropriate practice) represent the smaller discordant, ignorant but compliant subgroup (*n* = 31, 8.6%). The concentration of points in the lower right quadrant visually demonstrates that knowledge alone is insufficient to ensure appropriate behavior

The size of the discordant knowledgeable but non-compliant group was 87 participants, representing 24.1% of the total sample and 39.4% of the knowledgeable subgroup. Figure 2 provides a visual representation of this discordance, plotting individual participant knowledge scores against practice scores and clearly showing the concentration of points in the lower right quadrant. Table 6 presents the concordance and discordance matrix.

**Table 6:** Knowledge-practice concordance and discordance among Suez University students (*N* = 361)

	Appropriate Practice (*n* = 165)	Inappropriate Practice (*n* = 196)	Total
Adequate Knowledge (*n* = 221)	134 (60.6%)	87 (39.4%)	221
Inadequate Knowledge (*n* = 140)	31 (22.1%)	109 (77.9%)	140
Total	165	196	361

Discordance was defined as adequate knowledge (score ≥ 60%) combined with inappropriate practice (score below the median, which was 54.2). Values are presented as n (%). The discordant knowledgeable but non-compliant group (adequate knowledge + inappropriate practice) comprised 87 participants (24.1% of the total sample, 39.4% of knowledgeable students). The discordant, ignorant but compliant group (inadequate knowledge + appropriate practice) comprised 31 participants (8.6% of the total sample).

### Sources of antibiotic information and their associations

Participants were asked to identify their primary source of antibiotic information, defined as the source they use most frequently or rely upon most heavily when making antibiotic-related decisions. Of the 361 participants, 38 (10.5%) reported two or more equally frequent sources and required the ‘most trusted’ criterion to determine their primary source. Physicians were the most common primary source, reported by 145 participants (40.2%). The internet and social media were the primary source for 98 participants (27.1%). Pharmacists were the primary source for 56 participants (15.5%). Family members were the primary source for 42 participants (11.6%), and friends for 20 participants (5.5%).

Strikingly, students whose primary source was social media or family had substantially lower knowledge scores compared to those whose primary source was a physician. The mean knowledge score among students with social media as their primary source was 54.7% (SD = 15.2) versus 71.2% (SD = 12.4) among those with a physician as their primary source (*p* < 0.001). Similarly, the prevalence of inappropriate practices was markedly higher among students with social media as their primary source (65.3%) compared to those with a physician as their primary source (41.4%, *p* < 0.001). Table 7 presents the primary sources of antibiotic information and the associated knowledge and practice outcomes.

**Table 7:** Primary source of antibiotic information and associated knowledge and practice outcomes (*N* = 361)

Primary Information Source	Participants *n* (%)	Mean Knowledge Score % (SD)	*p*-value*	Proportion with Inappropriate Practice *n* (%)	*p*-value*
Physician	145 (40.2)	71.2 (12.4)	Reference	60 (41.4)	Reference
Pharmacist	56 (15.5)	64.2 (14.1)	0.002	31 (55.4)	0.04
Internet and social media	98 (27.1)	54.7 (15.2)	< 0.001	64 (65.3)	< 0.001
Family	42 (11.6)	52.3 (14.8)	< 0.001	28 (66.7)	< 0.001
Friends	20 (5.5)	56.1 (15.3)	< 0.001	13 (65.0)	0.002

*Compared to the physician as the primary source (reference category). Independent t-tests were used for knowledge score comparisons, justified by the normal distribution of the knowledge variable (Shapiro-Wilk *p* = 0.23). Chi-square tests were used for practice proportion comparisons. The primary source was defined as the participant’s self-reported main source of antibiotic-related information. For participants who reported two or more equally frequent sources (*n* = 38, 10.5%), the ‘most trusted’ source was used as the primary source.

### Predictors of adequate knowledge, positive attitudes, and appropriate practices

Multivariable logistic regression identified independent predictors for each outcome. For adequate knowledge, female gender (aOR = 2.34, 95% CI: 1.48 to 3.71, *p* < 0.001), medical faculty enrollment (aOR = 4.12, 95% CI: 2.31 to 7.34, *p* < 0.001), and advanced academic year (third year or above) (aOR = 1.89, 95% CI: 1.21 to 2.95, *p* = 0.005) were significant positive predictors. Prior antibiotic use within the past 12 months was negatively associated with adequate knowledge (aOR = 0.58, 95% CI: 0.35 to 0.96, *p* = 0.03), suggesting that exposure to antibiotics might correlate with, or perhaps contribute to, misunderstanding.

Gender was not independently associated with attitudes after adjustment for other variables. Older students (23 to 28 years) were independently more likely to hold positive attitudes compared to the youngest age group (aOR = 1.98, 95% CI: 1.12 to 3.52, *p* = 0.02), a finding consistent with the general observation that health-related attitudes mature with age and accumulated healthcare experience.

**Fig. 3:**
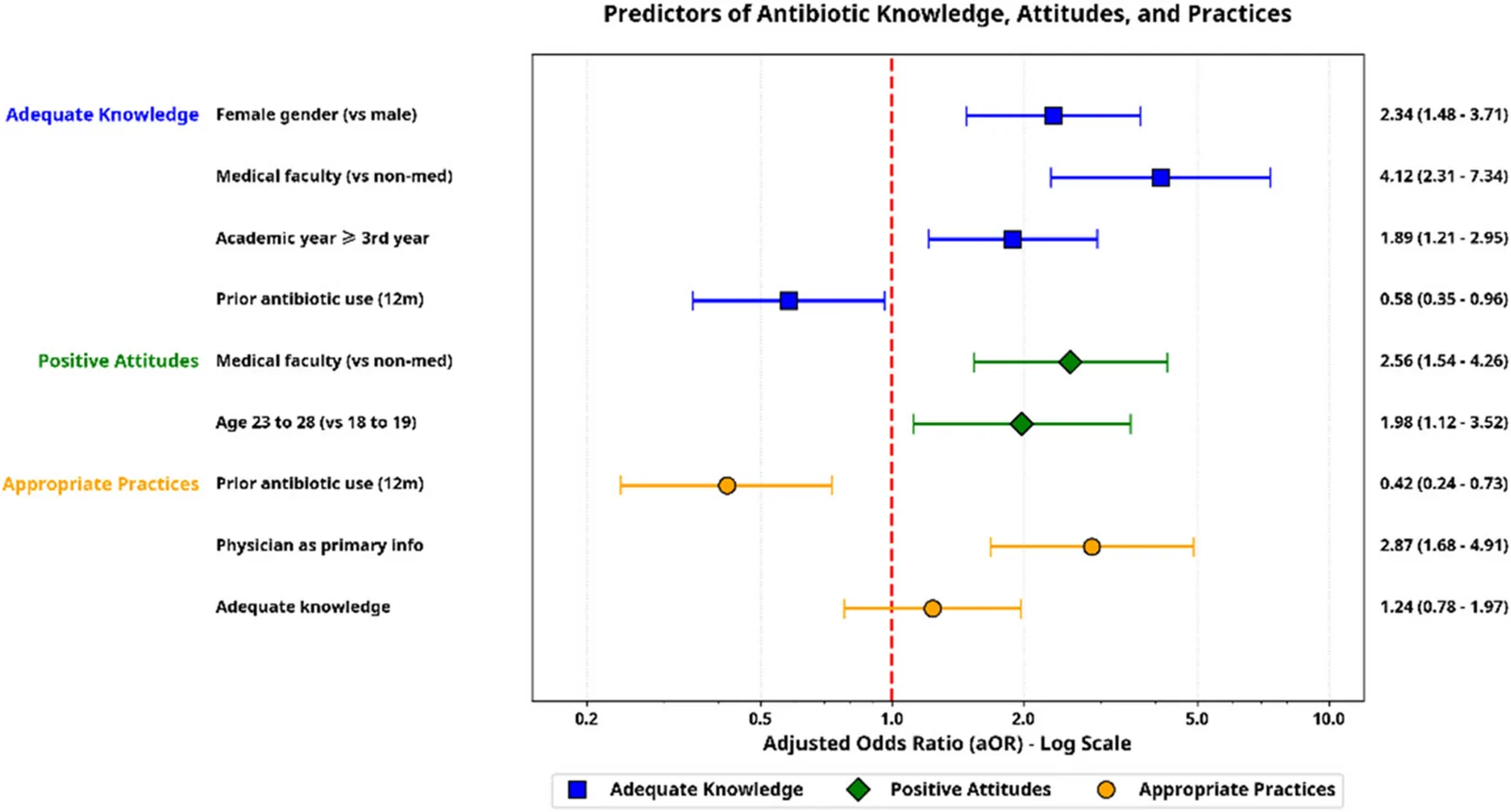
Forest plot of adjusted odds ratios (aOR) with 95% confidence intervals for predictors of adequate knowledge (blue squares), positive attitudes (green diamonds), and appropriate practices (orange circles). The vertical dashed line at aOR = 1 represents no effect. Predictors to the right of this line (aOR > 1) are positively associated with the outcome; predictors to the left (aOR < 1) are negatively associated. Female gender and medical faculty strongly predicted adequate knowledge. Prior antibiotic use within the past 12 months negatively predicted both knowledge and practices, but the effect was substantially stronger for practices (aOR = 0.42) than for knowledge (aOR = 0.58). Adequate knowledge did not independently predict appropriate practices (aOR = 1.24, 95% CI crosses 1.0), confirming the discordance identified in the primary analysis. The x-axis is shown on a log scale

For appropriate practices, the regression model revealed a different set of predictors. Prior antibiotic use within the past 12 months was the strongest negative predictor (aOR = 0.42, 95% CI: 0.24 to 0.73, *p* = 0.002). Having a physician as a primary information source was positively associated with appropriate practices (aOR = 2.87, 95% CI: 1.68 to 4.91, *p* < 0.001). Notably, adequate knowledge was not independently associated with appropriate practices after adjustment for other variables (aOR = 1.24, 95% CI: 0.78 to 1.97, *p* = 0.36) and was therefore not retained in the final model. Table 8 and Fig. 3 present the multivariable regression results.

Complete-case analysis for the logistic regression models included the following numbers of participants: adequate knowledge model (*n* = 347), positive attitudes model (*n* = 361), appropriate practices model (*n* = 347), and discordance model (*n* = 347). The positive attitudes model included all 361 participants because its predictor variables (medical faculty and age) had complete data for all participants.

**Table 8:** Multivariable logistic regression predictors of adequate knowledge, positive attitudes, and appropriate practices

Outcome	Predictor	aOR	95% CI	*p*-value
Adequate Knowledge				
	Female gender (vs. male)	2.34	1.48 to 3.71	< 0.001
	Medical faculty (vs. non-medical)	4.12	2.31 to 7.34	< 0.001
	Academic year ≥ third year (vs. first/second)	1.89	1.21 to 2.95	0.005
	Prior antibiotic use (past 12 months)	0.58	0.35 to 0.96	0.03
Positive Attitudes				
	Medical faculty (vs. non-medical)	2.56	1.54 to 4.26	< 0.001
	Age 23 to 28 years (vs. 18 to 19)	1.98	1.12 to 3.52	0.02
Appropriate Practices				
	Prior antibiotic use (past 12 months)	0.42	0.24 to 0.73	0.002
	Physician as primary information source	2.87	1.68 to 4.91	< 0.001

Variables with *p* < 0.20 in univariable analysis were entered into each multivariable model. The final models include only predictors that were statistically significant (*p* < 0.05) and retained in the final parsimonious model. For appropriate practices, adequate knowledge was entered into the initial multivariable model but was not statistically significant (aOR = 1.24, 95% CI: 0.78 to 1.97, *p* = 0.36) and was therefore not retained in the final model. Model fit statistics: For the adequate knowledge model, Hosmer-Lemeshow χ² = 6.23, *p* = 0.62; AUC = 0.78 (95% CI: 0.73 to 0.83). For the positive attitudes model, Hosmer-Lemeshow χ² = 5.87, *p* = 0.66; AUC = 0.74 (95% CI: 0.69 to 0.79). For the appropriate practices model, Hosmer-Lemeshow χ² = 4.92, *p* = 0.77; AUC = 0.81 (95% CI: 0.76 to 0.86)

*aOR *adjusted odds ratio, *CI *confidence interval

### Predictors of knowledge-practice discordance (knowledgeable but non-compliant subgroup)

The dependent variable was membership in the discordant group (*n* = 87) versus all other participants (*n* = 274).

Prior antibiotic use within the past 12 months was one of the strongest independent predictors of discordance, with an adjusted odds ratio of 2.87 (95% CI: 1.52 to 5.42, *p* = 0.001), comparable in magnitude to the effect of social media as an information source (aOR = 2.23). Students who had taken antibiotics in the past year were nearly three times more likely to belong to the knowledgeable but non-compliant group (aOR = 2.87, 95% CI: 1.52 to 5.42, *p* = 0.001). This finding applies specifically to the subgroup of students with adequate knowledge (*n* = 221), not to the full sample. The large magnitude (aOR = 2.87) indicates that among students who know the correct information, those with prior antibiotic experience are nearly three times more likely to be discordant (knowledgeable but non-compliant) compared to those without prior use.

Female gender was also independently associated with discordance (aOR = 1.78, 95% CI: 1.04 to 3.05, *p* = 0.04). To explore whether the gender paradox could be explained by differential effects of other predictors, we tested interaction terms between gender and prior antibiotic use (*p* = 0.19) and between gender and primary information source (gender × social media, *p* = 0.27; gender × physician, *p* = 0.34). Neither interaction was statistically significant, indicating that the female discordance effect persists independently of these factors.

**Fig. 4:**
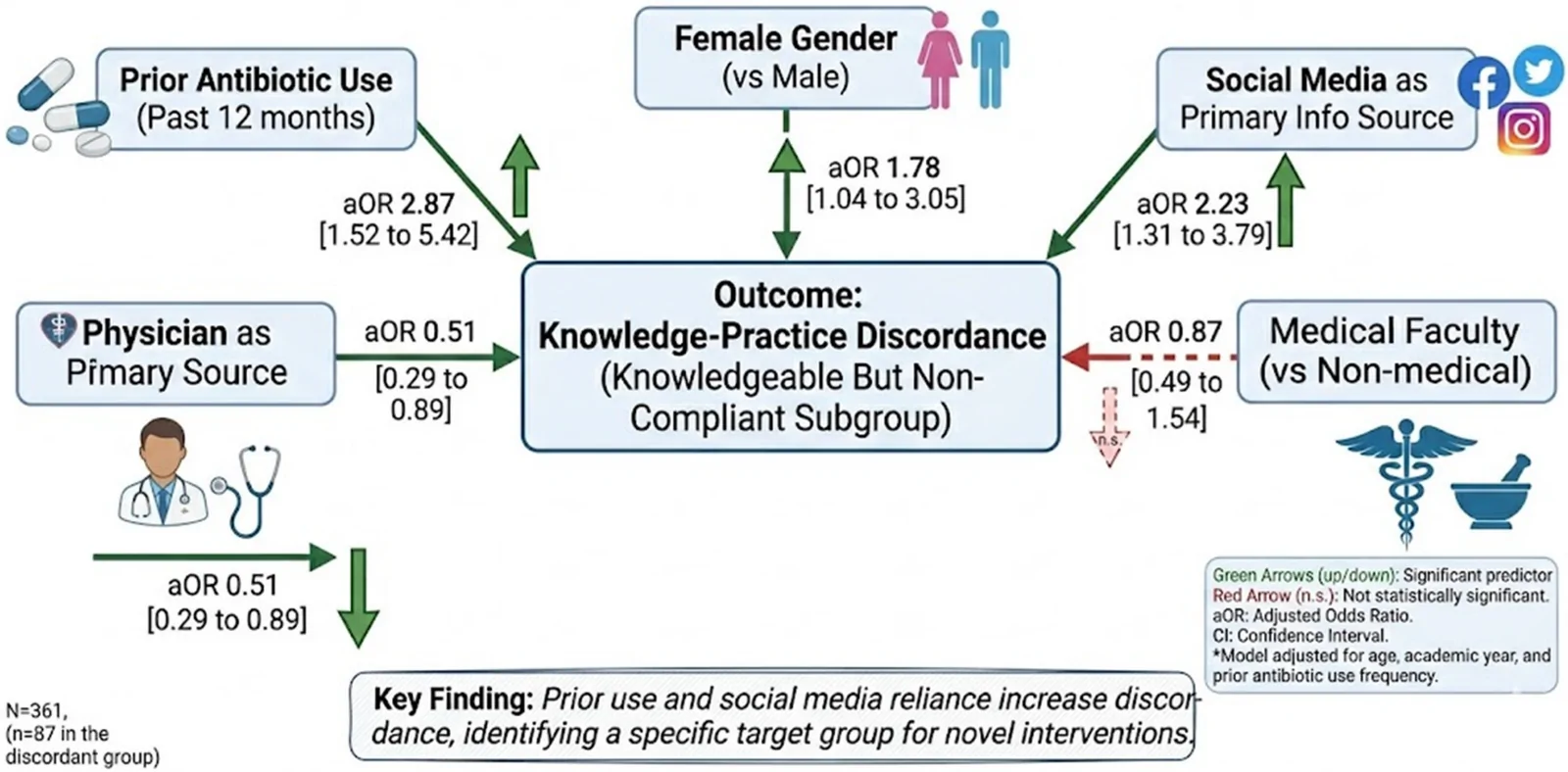
Directed acyclic graph illustrating predictors of knowledge-practice discordance (knowledgeable but non-compliant subgroup) among Suez University students (*N* = 361, discordant group *n* = 87). Numbers adjacent to arrows represent adjusted odds ratios (aOR) with 95% confidence intervals from multivariable logistic regression. Green arrows indicate statistically significant predictors (*p* < 0.05). The red arrow indicates a non-significant predictor (*p* = 0.63). Prior antibiotic use (aOR = 2.87) and social media reliance (aOR = 2.23) were the strongest positive predictors. Female gender (aOR = 1.78) was also positively associated. Physician as primary source (aOR = 0.51) was protective. Medical faculty enrollment was not independently associated (aOR = 0.87, 95% CI crosses 1.0). The model was adjusted for age, academic year, and prior antibiotic use frequency

Reliance on social media as a primary information source was associated with increased odds of discordance (aOR = 2.23, 95% CI: 1.31 to 3.79, *p* = 0.003). Conversely, having a physician as a primary information source was protective against discordance (aOR = 0.51, 95% CI: 0.29 to 0.89, *p* = 0.02). The adjusted associations between predictors and knowledge-practice discordance are illustrated in Fig. 4, while detailed regression coefficients are presented in Table 9.

Medical faculty enrollment was not associated with discordance after adjustment (aOR = 0.87, 95% CI: 0.49 to 1.54, *p* = 0.63), indicating that despite their superior knowledge, medical students were equally likely to be in the discordant group when other factors were accounted for. This is a sobering finding for medical education. Table 9 presents the predictors of discordance.

**Table 9:** Multivariable logistic regression predictors of knowledge-practice discordance (knowledgeable but non-compliant subgroup)

Predictor	aOR	95% CI	*p*-value
Prior antibiotic use (past 12 months)	2.87	1.52 to 5.42	0.001
Female gender (vs. male)	1.78	1.04 to 3.05	0.04
Social media as a primary information source	2.23	1.31 to 3.79	0.003
Physician as primary information source	0.51	0.29 to 0.89	0.02
Medical faculty (vs. non-medical)	0.87	0.49 to 1.54	0.63

Dependent variable: membership in the discordant knowledgeable but non-compliant group (*n* = 87) versus all other participants (*n* = 274). The model adjusted for age, academic year, and prior antibiotic use frequency. A *p*-value <0.05 was considered statistically significant. Model fit statistics: Hosmer-Lemeshow χ² = 5.31, *p* = 0.72; AUC = 0.79 (95% CI: 0.73 to 0.85)

*aOR *adjusted odds ratio, *CI *confidence interval

### Latent class analysis reveals four distinct behavioral profiles

The LCA identified four distinct classes of students based on their response patterns across knowledge, attitude, and practice items. Fit indices supported the four-class solution as optimal: the AIC (2847.6) and BIC (2912.3) were substantially lower than for the three-class solution (AIC = 2968.4, BIC = 3014.7) and only marginally higher than for the five-class solution (AIC = 2831.2, BIC = 2918.6), which had two classes that were not interpretable. The entropy for the four-class solution was 0.84, indicating excellent classification accuracy.Complete model fit indices for one to five class solutions are presented in Table 10.

**Fig. 5:**
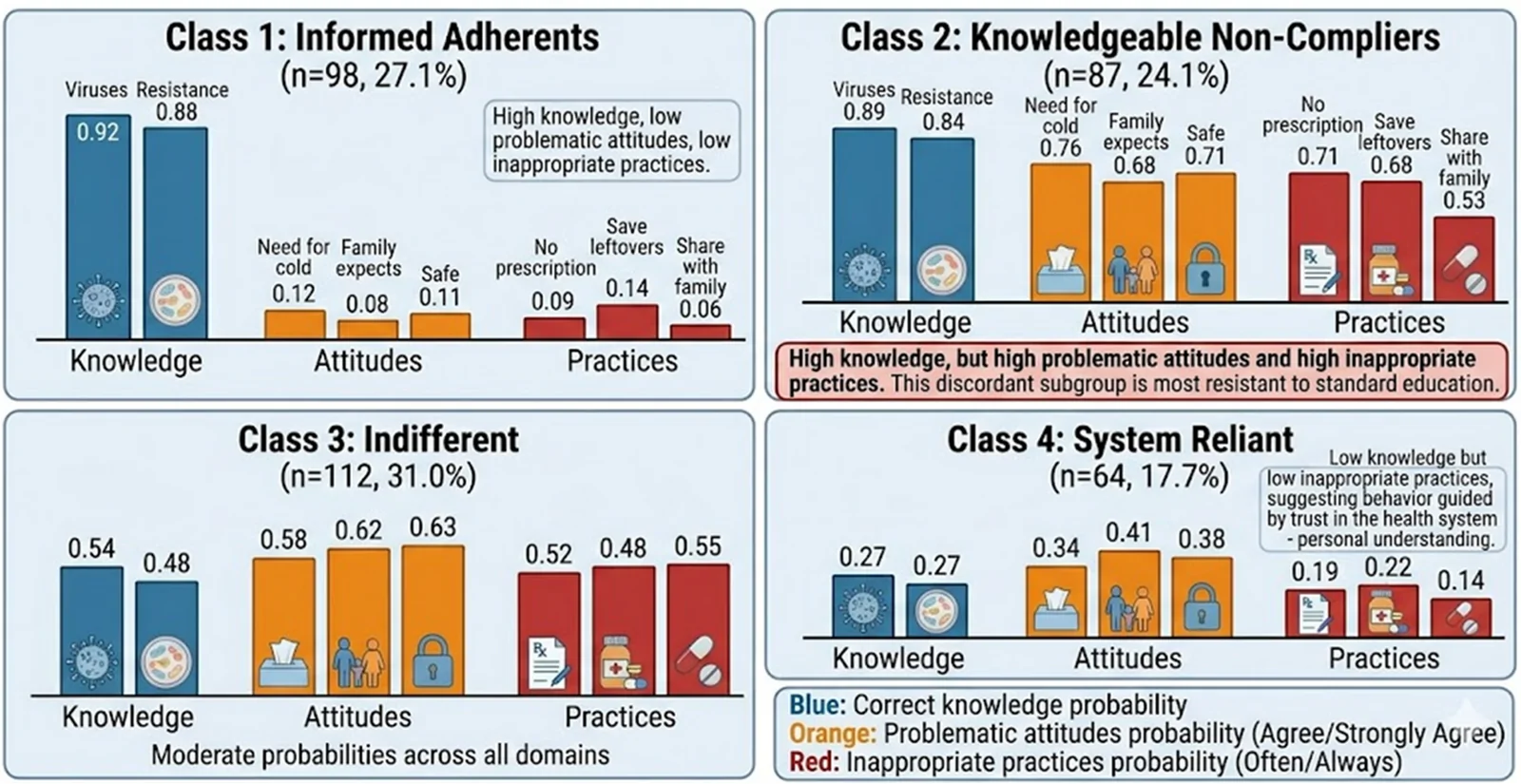
Latent class profiles of antibiotic related behaviors among Suez University students (*N* = 361). Each panel represents one of the four identified classes. Bars represent the probability of correct knowledge (blue), problematic attitudes (orange), and inappropriate practices (red). Class 1 (Informed Adherents, 27.1%) shows high knowledge, low problematic attitudes, and low inappropriate practices. Class 2 (Knowledgeable Non Compliers, 24.1%) shows high knowledge but high problematic attitudes and high inappropriate practices. This discordant subgroup is most resistant to standard education. Class 3 (Indifferent, 31.0%) shows moderate probabilities across all domains. Class 4 (System Reliant, 17.7%) shows low knowledge but low inappropriate practices, suggesting behavior guided by trust in the health system rather than personal understanding

Class 1: The Informed Adherents (n = 98, 27.1%). Students in this class demonstrated high probability of correct knowledge responses (above 80% for all knowledge items), favorable attitudes (low probability of endorsing problematic beliefs), and appropriate practices (low probability of self-medication or sharing). This class represents the target behavior profile that interventions aim to achieve. Class 2: The Knowledgeable Non-Compliers (n = 87, 24.1%). This class showed high probability of correct knowledge responses (comparable to Class 1) but high probability of endorsing problematic attitudes (particularly the belief that antibiotics speed cold recovery, probability 0.76) and high probability of inappropriate practices (non-prescription use probability 0.71, leftover storage probability 0.68). Notably, Class 2 (n = 87) corresponds exactly to the discordant knowledgeable but non-compliant subgroup identified in the primary discordance analysis (n = 87, Table 6), providing convergent validity for both analytical approaches. This class corresponds to the discordant subgroup of students who demonstrate adequate knowledge yet report inappropriate practices. Class 3: The Indifferent (n = 112, 31.0%). Students in this class had moderate probabilities of correct knowledge (ranging from 0.45 to 0.65 across items), neutral attitudes (neither strongly favorable nor strongly unfavorable), and inconsistent practices. They represent the largest single class and may be the most amenable to standard educational interventions, as they have not yet developed strongly entrenched counterproductive beliefs. Class 4: The System Reliant (n = 64, 17.7%). This class had a low probability of correct knowledge (below 0.40 for all items) but paradoxically a high probability of appropriate practices (non-prescription use probability 0.19, sharing probability 0.14). These students appear to behave correctly, not because they understand why, but because they trust physicians or follow family practices. They represent a different kind of discordance: appropriate behavior without underlying knowledge. Conceptually, this class overlaps with and expands upon the ‘ignorant but compliant’ subgroup identified in Table 6 (*n* = 31), though Class 4 is larger (*n* = 64). The larger size of Class 4 suggests that the LCA, using multiple indicator variables, identified additional students who practice appropriately without adequate knowledge beyond those captured by the simpler discordance matrix. Table 11 presents the item response probabilities for each class alongside the gender distribution across classes, and Fig. 5 visualizes the class profiles.

To further explore the female paradox, cross-tabulation of LCA class membership by gender was examined (Table 11). Female students were disproportionately represented in Class 2 (Knowledgeable Non-Compliers), comprising 58 of the 87 participants in this class (66.7%), compared to 29 males (33.3%). This pairwise difference for Class 2 compared to the other classes combined was statistically significant (χ² = 4.21, *p* = 0.04). In contrast, Class 1 (Informed Adherents) had near equal gender distribution (51 females, 47 males). These findings indicate that the discordant profile (high knowledge, problematic attitudes, inappropriate practices) is significantly more common among female students, directly operationalizing the female paradox observed in the comparative analysis.

Beyond the gender distribution, the behavioral profiles identified through LCA map systematically onto the Theory of Planned Behavior framework. The four behavioral profiles identified through LCA can be mapped onto the Theory of Planned Behavior (TPB) framework introduced in the Introduction. Class 1 (Informed Adherents) represents the TPB target state: favorable attitudes, strong subjective norms supporting appropriate use, and perceived behavioral control enabling adherence to guidelines. Class 2 (Knowledgeable Non-Compliers) represents a TPB failure mode: negative attitudes (e.g., belief that antibiotics speed cold recovery) and perceived subjective norms (family pressure) override factual knowledge, leading to inappropriate practices despite adequate understanding. Class 3 (Indifferent) reflects ambivalence across TPB domains: neither strongly favorable nor unfavorable attitudes, neutral subjective norms, and inconsistent perceived behavioral control. Class 4 (System Reliant) maps to a pattern of strong subjective norm compliance (trust in physicians or family) in the absence of personal attitudes or knowledge, representing a fragile form of appropriate behavior. Notably, perceived behavioral control (ease of obtaining antibiotics without a prescription) was not directly measured in this study, representing a scope limitation that future research should address.

**Table 10:** Latent Class Model Fit Indices for One to Five Class Solutions

Number of Classes	AIC	BIC	aBIC	Entropy	Interpretability
1	3245.6	3289.4	3251.2	NA	Too few classes
2	3087.3	3142.8	3089.6	0.72	Insufficient separation
3	2968.4	3014.7	2968.3	0.78	Moderate fit
4	2847.6	2912.3	2847.6	0.84	Optimal fit (selected)
5	2831.2	2918.6	2831.2	0.81	Two classes uninterpretable

Lower AIC, BIC, and aBIC values indicate better model fit. Entropy values closer to 1 indicate better classification accuracy. The four-class solution was selected based on optimal fit indices, highest entropy, and interpretability of all four classes

*AIC *Akaike Information Criterion, *BIC *Bayesian Information Criterion, *aBIC *sample size adjusted BIC

**Table 11:** Item response probabilities by latent class (*N* = 361)

Indicator Variable	Class 1 Informed Adherents (*n* = 98)	Class 2 Knowledgeable Non-Compliers (*n* = 87)	Class 3 Indifferent (*n* = 112)	Class 4 System Reliant (*n* = 64)
Knowledge items (probability correct)				
Viruses not killed by antibiotics	0.92	0.89	0.54	0.31
Resistance from unnecessary use	0.88	0.84	0.48	0.27
Attitude items (probability agree/strongly agree)				
Need antibiotics for cold recovery	0.12	0.76	0.58	0.34
Family expects antibiotic use	0.08	0.68	0.62	0.41
Antibiotics are completely safe	0.11	0.71	0.63	0.38
Practice items (probability often/always)				
Take antibiotics without prescription	0.09	0.71	0.52	0.19
Save leftovers for future use	0.14	0.68	0.55	0.22
Share antibiotics with family	0.06	0.53	0.48	0.14
Gender distribution *n* (%)				
Female	51 (52.0%)	58 (66.7%)	58 (51.8%)	31 (48.4%)
Male	47 (48.0%)	29 (33.3%)	54 (48.2%)	33 (51.6%)

Item response probabilities represent the probability of each indicator being endorsed within each latent class. Overall gender distribution across all four classes was significantly different (χ² = 8.94, df = 3, *p* = 0.03). Pairwise comparison for Class 2 versus all other classes combined: χ² = 4.21, *p* = 0.04

### Mediation analysis confirms the attitude pathway

Mediation analysis was conducted to test whether attitudes mediate the relationship between knowledge and practices. Practice was treated as a continuous score (0 to 100 scale) to preserve the full range of variation. The total effect of knowledge on practices was statistically significant (β = 0.31, 95% CI: 0.19 to 0.43, *p* < 0.001), indicating that higher knowledge scores were associated with more appropriate practices before accounting for attitudes. Although knowledge showed a significant unadjusted association with practices, this association was not independent after adjustment, suggesting mediation or confounding effects.

However, when attitudes were entered as a mediator, a different picture emerged. The direct effect of knowledge on practices, after controlling for attitudes and covariates, was substantially reduced and no longer statistically significant (β = 0.09, 95% CI: -0.04 to 0.22, *p* = 0.18). The indirect effect of knowledge on practices through attitudes was significant (β = 0.22, 95% CI: 0.14 to 0.31), accounting for 71.0% of the total effect.

**Fig. 6:**
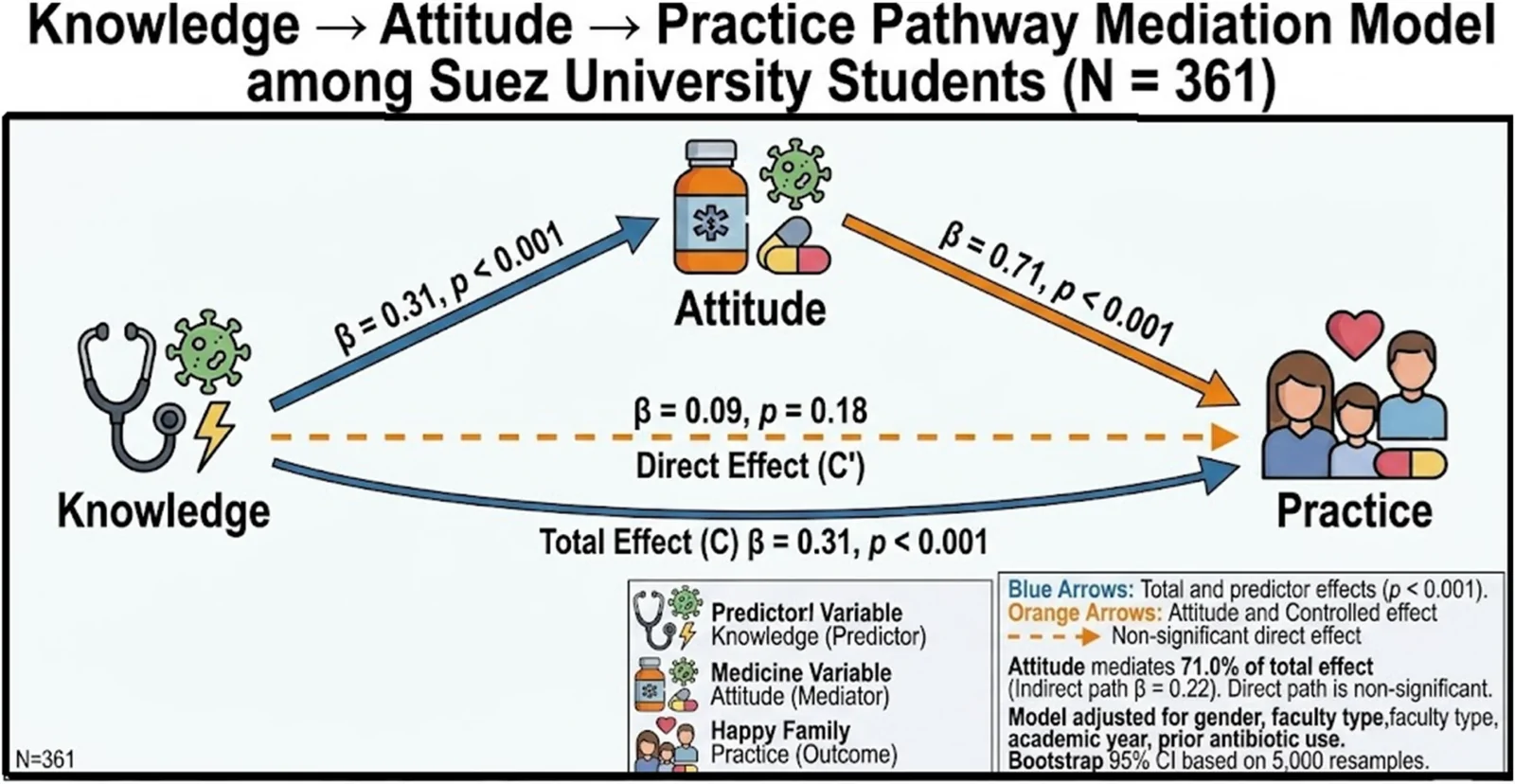
Path diagram of the mediation model examining the relationship between knowledge, attitudes, and practices among Suez University students (*N* = 361). Numbers represent standardized regression coefficients. The path from knowledge to practice (direct effect, β = 0.09, *p* = 0.18) is non-significant after accounting for attitudes. The indirect path through attitudes (knowledge → attitude → practice) is significant (β = 0.22, *p* < 0.001). Attitudes mediate 71% of the total effect of knowledge on practices. This statistical mediation is consistent with the hypothesis that the association between knowledge and behavior is statistically mediated by attitudes. This pattern corresponds to the observation that students with adequate knowledge but unfavorable attitudes continue to report antibiotic misuse. Covariates (gender, faculty type, academic year, prior antibiotic use) were included but are not shown for visual clarity

This finding provides statistical evidence consistent with the discordance mechanism, though causal interpretation is limited by the cross-sectional design. The direct association between knowledge and practices was no longer statistically significant after adjustment for attitudes. This pattern is consistent with statistical mediation, but alternative explanations cannot be ruled out. The practical implication, while requiring confirmation in longitudinal studies, is that interventions targeting knowledge alone may be insufficient if attitudes remain unaddressed. Table 12 presents the mediation analysis results, and Fig. 6 visualizes the pathway.

**Table 12:** Mediation analysis of knowledge → attitude → practice pathway

Effect	β coefficient	95% CI	*p*-value	Proportion of total effect
Total effect (knowledge → practice)	0.31	0.19 to 0.43	< 0.001	100%
Direct effect (knowledge → practice, controlling for attitude)	0.09	-0.04 to 0.22	0.18	29.0%
Indirect effect (knowledge → attitude → practice)	0.22	0.14 to 0.31	< 0.001	71.0%

Effects are reported as unstandardized coefficients. Bootstrap 95% confidence intervals were calculated using 5,000 resamples. Proportion mediated = indirect effect / total effect = 0.22 / 0.31 = 0.710 (71.0%). Results should be interpreted as associative due to the cross-sectional design. This proportion mediated estimate should be interpreted cautiously in cross-sectional designs, where the total effect may underestimate the true direct effect due to measurement timing. Adjusted for gender, faculty type, academic year, and prior antibiotic use. The indirect effect is significant because the confidence interval does not contain zero. The direct effect is not significant, a pattern consistent with statistical mediation given the cross-sectional design

*β *regression coefficient, *CI *confidence interval

### Sensitivity analyses

To assess the robustness of the discordance findings, two sensitivity analyses were conducted as prespecified in the Methods section. First, the adequate knowledge cutoff was varied from 60% to 50% and to 70%. Using the 50% cutoff, the proportion of students with adequate knowledge increased from 61.2% to 74.5% (269 participants), and the discordant knowledgeable but non-compliant subgroup increased from 87 to 103 participants (28.5% of the total sample). Using the 70% cutoff, the proportion with adequate knowledge decreased to 43.8% (158 participants), and the discordant subgroup decreased to 64 participants (17.7% of the total sample). In both sensitivity definitions, the pattern of predictors for discordance remained consistent: prior antibiotic use, female gender, and social media reliance remained significant positive predictors (*p* < 0.05 for all), while physician as primary source remained protective (*p* < 0.05).

Second, the median practice cutoff for defining inappropriate practice was replaced with the 25th percentile (practice score ≤ 45.8), representing a stricter definition of non-compliance that captures only the most extreme inappropriate practices. Under this more extreme definition, the discordant knowledgeable but non-compliant subgroup comprised 49 participants (13.6% of the total sample). The direction and significance of all predictors remained unchanged, though effect sizes were attenuated (prior antibiotic use aOR = 2.15, 95% CI: 1.18 to 3.92, *p* = 0.01). These sensitivity analyses confirm that the primary discordance findings are robust to alternative cutoff definitions.

## Discussion

Antimicrobial resistance has been described as a silent pandemic, and Egypt is among the countries most heavily affected. The Global Burden of Disease study estimated that in 2019, Egypt recorded 16,100 deaths directly attributable to AMR and 56,600 deaths associated with resistant infections, ranking the country 147th out of 204 in age-standardized mortality rates from resistance [4]. These figures reflect not only clinical prescribing patterns but also community-level behaviors, including self-medication, antibiotic sharing, and the widespread belief that antibiotics are harmless recovery aids for viral illnesses. University students occupy a critical position in this landscape. They are old enough to make independent health decisions, yet young enough that their behaviors are still modifiable. They are also future professionals, including prescribers, who will shape antibiotic use for decades. Understanding the specific drivers of misuse in this population is therefore not an academic exercise but a prerequisite for effective stewardship. The present study was designed to move beyond descriptive KAP reporting and to quantify, through discordance analysis, latent class profiling, and mediation testing, the mechanisms that separate knowledge from action.

The study sample reflected the gender distribution of Suez University, with females comprising 54.8% of participants (Table 1). The majority of students (79.2%) reported using antibiotics within the past 12 months, a behavioral exposure rate substantially higher than the 35.1% good-awareness rate reported by Mostafa and colleagues [11], noting that these reflect different outcomes: actual use versus knowledge-based awareness. This discrepancy likely reflects differences in sampling, as our study included medical students who may have higher exposure to antibiotics through clinical training or family expectations, but it may also indicate genuine regional or temporal differences in antibiotic consumption. More concerning is the source of these antibiotics. Among those who reported prior use, only 39.2% obtained them through a physician’s prescription. The remainder obtained antibiotics directly from a pharmacy without a prescription (34.3%), used leftover antibiotics from previous illnesses (16.8%), or received them from family or friends (9.8%). These figures are consistent with a study from Ghana, where 40.2% of university students reported obtaining antibiotics from pharmacies without a prescription, and with research from Jordan, where 60.7% of students self-medicated with antibiotics [10, 38]. The implication is that the regulatory framework requiring prescriptions is widely circumvented. Students who can obtain antibiotics without professional consultation are unlikely to develop the habit of seeking medical advice for minor infections, and the absence of gatekeeping removes a natural opportunity for education.

The mean knowledge score of 62.4% places Suez University students in the moderate range compared to other LMIC university populations. A study from Malaysia reported mean knowledge scores of 58.3% among non-medical students, while a study from Pakistan found that 68.7% of students had adequate knowledge using a similar cutoff [39, 40]. The variation across studies likely reflects differences in questionnaire design, sampling, and local antibiotic access policies. More informative than the overall mean is the pattern of specific misconceptions. Only 45.2% of students correctly understood that antibiotics are ineffective against viral infections (Table 2). This is not a trivial knowledge gap. The common cold and viral pharyngitis account for the majority of outpatient antibiotic prescriptions globally, and patient demand is a well-documented driver of unnecessary prescribing [41]. A student who believes that antibiotics will shorten a cold is likely to request them, and a busy clinician facing time pressure may comply.

The low correct response rate for the prescription requirement item (49.3%) is particularly telling. Students cannot know that antibiotics require a prescription if their lived experience is that pharmacies dispense them freely. This finding suggests that individual knowledge is shaped by structural reality. In settings where enforcement of prescription laws is weak, knowledge about the law becomes irrelevant to behavior. A qualitative study from Egypt found that pharmacists often dispense antibiotics without a prescription because they perceive patient demand as unavoidable and fear losing business to competitors [5]. The knowledge gap we observed may therefore be less a failure of education and more a rational adaptation to an environment where prescriptions are not routinely required.

The attitude data reveal a consistent pattern of beliefs that favor inappropriate use. Over half of students endorsed the statement that antibiotics are needed to recover quickly from a cold (56.2%), and 43.5% agreed that antibiotics are completely safe (Table 3). These beliefs are not independent; they form a cognitive framework in which antibiotics are perceived as low-risk, high-benefit tools for symptom relief. The Theory of Planned Behavior, introduced in the Introduction, posits that behavioral intention is shaped by attitudes toward the behavior, subjective norms, and perceived behavioral control [12]. Our findings support this framework. The belief that antibiotics speed recovery (attitude) and the perception that family expects antibiotic use (subjective norm) both predicted discordance in the regression models. Notably, 51.5% of students agreed that their family expects them to take antibiotics when sick. This is not merely a personal preference. It is a social expectation embedded in household routines. A student who refuses antibiotics may face pressure not only from their own symptoms but from family members who equate antibiotic use with good care.

The mediation analysis (Table 12) provides quantitative evidence for the pathway from knowledge to practice through attitudes. The indirect effect of knowledge on practices via attitudes was significant (β = 0.22), while the direct effect was not (β = 0.09, *p* = 0.18). Attitudes statistically accounted for 71% of the total association between knowledge and practices. This pattern is consistent with cross-sectional mediation, though causal interpretation would require longitudinal data. The finding aligns with research from the United Arab Emirates, where attitudes were stronger predictors of antibiotic self-medication than knowledge, and with a meta-analysis of health behavior change interventions, which found that attitude modification was more effective than knowledge transfer alone for reducing risky health behaviors [12, 42].

In the multivariable analysis, medical faculty enrollment was the strongest predictor of positive attitudes (aOR = 2.56, 95% CI: 1.54 to 4.26, *p* < 0.001), which is expected given that medical students receive formal education on antimicrobial resistance and appropriate prescribing. More notably, older age (23 to 28 years) was independently associated with positive attitudes compared to the youngest age group (aOR = 1.98, 95% CI: 1.12 to 3.52, *p* = 0.02). This age gradient suggests that health-related attitudes toward antibiotic use mature with accumulated healthcare experience and increased autonomy from family norms. Older students, who are further into their academic training, may have greater personal agency in health decision-making or more exposure to the clinical consequences of antibiotic resistance, both of which could shape more favorable attitudes. Gender was not independently associated with attitudes after adjustment for other variables, indicating that the female paradox observed for discordance is not explained by attitudinal differences.

The discordance analysis identified 87 students (24.1% of the total sample) who possessed adequate knowledge yet reported inappropriate practices (Table 6). The quadrant plot (Fig. 2) shows these students concentrated in the lower right quadrant, where high knowledge coexists with poor practices. They are not ignorant. They are not forgetful. They are, in effect, knowledgeable non-compliers. The LCA independently identified a class of identical size (*n* = 87) with the same profile, labeled Class 2 (Knowledgeable Non-Compliers, Table 11). The convergent validity across two different analytical methods strengthens confidence that this subgroup is a genuine behavioral phenotype rather than a statistical artifact.

Beyond the discordant subgroup analysis, the absence of any significant difference in practice scores across all five faculty types (*p* = 0.18, Table 5) provides further evidence of the knowledge-practice disconnect. Despite knowledge scores ranging from 52.4% among humanities students to 74.3% among medical students, a gap of nearly 22% points, practice scores remained statistically indistinguishable across all faculties. This finding demonstrates that knowledge acquisition, whether from formal medical education or general university study, does not automatically translate into behavioral change. Even students who receive extensive training on antimicrobial resistance (medical students) report antibiotic practices no better than those of their peers with minimal formal health education (humanities students).

What distinguishes these students? The item response probabilities in Table 11 show that Class 2 has high knowledge (0.89 and 0.84 for the two knowledge items) but also high probabilities of endorsing problematic attitudes: need for antibiotics for cold recovery (0.76), family expectation (0.68), and safety misperception (0.71). Their practice probabilities are correspondingly high: taking antibiotics without prescription (0.71), saving leftovers (0.68), and sharing with family (0.53). In contrast, Class 1 (Informed Adherents) has similarly high knowledge but low probabilities on all attitude and practice items. The difference between Class 1 and Class 2 is not knowledge. It is attitudes. This pattern is consistent with the mediation analysis and directly supports the theoretical framework. Interventions that target knowledge alone will not reach Class 2. They already know the facts. Changing their behavior requires changing their beliefs about necessity, safety, and social norms.

Comparable behavioral typologies have been identified in other LMICs. A latent class analysis of antibiotic use among Chinese community residents identified four classes, including a “high knowledge risky practice” class analogous to our Knowledgeable Non-Compliers [43]. A study from Thailand using cluster analysis found that individuals with high knowledge but low perceived risk of resistance were the most likely to self-medicate [44]. The convergence of findings across different LMICs suggests that the knowledgeable non-complier phenotype is not unique to Egypt. It may represent a general failure mode of educational interventions that assume knowledge deficits are the primary barrier.

Class 4 (System Reliant, 17.7% of the sample) represents a different challenge. These students have low knowledge (probabilities 0.31 and 0.27) but report appropriate practices (non-prescription use probability 0.19, sharing probability 0.14). Their appropriate behavior appears to depend on external guidance from physicians or family. This is fragile. If their trusted source provides incorrect advice or becomes unavailable, their practices could deteriorate. The LCA identified nearly twice as many students in Class 4 (*n* = 64) as the simple discordance matrix identified as ignorant but compliant (*n* = 31). This discrepancy suggests that the LCA, using multiple indicator variables, captured additional students who practice appropriately without adequate knowledge. From a stewardship perspective, these students are not currently problematic, but they are vulnerable. Interventions that focus exclusively on knowledge building may neglect the need to reinforce system trust while simultaneously building personal understanding.

The relationship between prior antibiotic use and outcomes requires careful interpretation across the two regression models, as the denominators differ substantially. In Table 8, which models the full sample (*N* = 361), prior antibiotic use was associated with lower odds of appropriate practice (aOR = 0.42). This finding indicates that students who had taken antibiotics in the past year were generally less likely to report appropriate practices compared to those with no prior use. In contrast, Table 9 examines a different outcome: discordance among the subgroup of students with adequate knowledge (*n* = 221). Within this knowledgeable subgroup, prior antibiotic use was associated with higher odds of belonging to the discordant knowledgeable but non-compliant group (aOR = 2.87). These two findings are complementary rather than contradictory. Prior antibiotic use functions as a general risk factor for poor practices across the full sample. Among students who already possess adequate knowledge, prior use additionally identifies those who fail to translate that knowledge into action. The different magnitudes (0.42 vs. 2.87) reflect the different denominators and outcomes: the full sample includes students with inadequate knowledge who already have poor practices, whereas the knowledgeable subgroup isolates the discordance phenomenon. Notably, the aOR of 2.87 for prior antibiotic use in Table 9 is numerically identical to the aOR for physician as primary source in Table 8 (2.87 in both cases). This coincidence arises from rounding and the specific coefficients of each model; the two values refer to entirely different predictors, outcomes, and sample subsets. No direct comparison between these two aORs is intended or meaningful.

The novelty of this study lies not in the observation that knowledge-practice discordance exists, but in the methodological approach used to characterize it and the theoretical grounding provided for its mechanisms. To our knowledge, no published study from Egypt or the broader MENA region has simultaneously quantified knowledge-practice discordance using a formally defined composite outcome, applied latent class analysis to identify behavioral phenotypes, and tested the attitudinal mediation pathway using formal mediation analysis. Most previous KAP studies have reported descriptive statistics, compared mean scores across subgroups, and concluded that more education is needed. The present study moves beyond description in three specific ways. First, the discordance analysis explicitly identifies the 24.1% of students for whom education alone will fail. Second, the LCA provides a behavioral typology that assigns each student to a class with distinct implications for intervention. Third, the mediation analysis estimates the proportion of the Knowledge-practice association that operates through attitudes (71%), providing a quantitative target for intervention design. Additionally, the convergent identification of the same *n* = 87 discordant subgroup by two independent analytical approaches provides cross-method validation rarely demonstrated in KAP research, and the formal characterization of a System Reliant behavioral phenotype represents a novel contribution to the taxonomy of antibiotic use behaviors, with distinct implications for the fragility of appropriate practice in low-enforcement healthcare environments. These analytical innovations, applied to a rigorously sampled population of 361 students across 16 faculties, represent a substantial advancement over the existing Egyptian and regional literature.

Female students had higher knowledge scores (66.2% vs. 58.1%) and higher attitude scores (median 61.5 vs. 55.4) than males, yet they were more likely to belong to the discordant knowledgeable but non-compliant subgroup (aOR = 1.78, Table 9) and did not have significantly better practices (Table 5). This female paradox is not an artifact of measurement. The gender distribution across LCA classes (Table 11) shows that females constitute 66.7% of Class 2 (Knowledgeable Non-Compliers) but only 52.0% of Class 1 (Informed Adherents). Females who know the facts and hold favorable attitudes are still more likely to misuse antibiotics.

Several explanations are plausible, though our data cannot definitively distinguish among them. Formal interaction testing revealed that the effect of gender on discordance was not moderated by prior antibiotic use or information source, suggesting the female paradox operates through mechanisms not captured by the measured predictors. Qualitative research exploring gender-specific social expectations and healthcare access patterns would be valuable to elucidate this finding. First, subjective norms may operate differently for females. In many Egyptian households, women are primarily responsible for family health decisions, including caring for sick children and elderly relatives [45]. A female student who knows that antibiotics are not needed for her own cold may still request them because her family expects her to model appropriate illness behavior. Second, females may have different access to healthcare resources. They may be more likely to accompany family members to clinics, creating more opportunities for obtaining prescriptions or leftover antibiotics. Similar gender patterns have been reported elsewhere. A study from Malaysia found that female university students had higher knowledge but also higher rates of antibiotic self-medication [46]. Research from Ethiopia reported that females were more likely to store leftover antibiotics, and a study from India found that mothers with higher education (a proxy for knowledge) were paradoxically more likely to administer antibiotics for childhood fevers [47, 48]. The female paradox is not a failure of female students. It is a failure of interventions that assume knowledge and attitudes are sufficient. Future interventions targeting females should address the social contexts in which women make health decisions.

The association between primary information source and outcomes is one of the clearest findings of this study (Table 7). Students whose primary source was a physician had mean knowledge scores of 71.2% and an inappropriate practice prevalence of 41.4%. Students whose primary source was social media had scores of 54.7% and 65.3%, respectively. The differences are not small. They represent a gap of 16.5% points in knowledge and 23.9% points in inappropriate practice prevalence. Social media as a primary source was the second strongest predictor of discordance (aOR = 2.23), of similar magnitude to prior antibiotic use (aOR = 2.87), with overlapping confidence intervals indicating no statistically significant difference between the two effect sizes.

Notably, students who identified pharmacists as their primary information source occupied an intermediate position between physicians and social media. Their mean knowledge score (64.2%, *p* = 0.002 vs. physician) and inappropriate practice prevalence (55.4%, *p* = 0.04 vs. physician) were significantly worse than those of physician-reliant students, but better than those of social media-reliant students (54.7% knowledge, 65.3% inappropriate practice) (Table 7). This gradient suggests that pharmacists provide partial but incomplete antibiotic education. In Egypt, where prescription enforcement is weak and 34.3% of students in our sample obtained antibiotics directly from pharmacies without a prescription, the pharmacist is effectively the primary healthcare contact for many young adults. Engaging pharmacists in antimicrobial stewardship education, as recommended by the WHO AWaRe framework, could leverage this existing touchpoint to reduce inappropriate access while improving patient knowledge.

Why does social media reliance associate with worse outcomes? The mechanisms are likely multifactorial. First, social media platforms do not prioritize accuracy. They prioritize engagement. Content that is emotionally compelling, extreme, or reassuring often spreads faster than nuanced, evidence-based information [49]. A post claiming that antibiotics cured someone’s cold quickly will receive more shares than a post explaining why antibiotics do not work for viruses. Second, social media algorithms create filter bubbles. A student who searches for antibiotic information once will receive repeated recommendations for similar content, reinforcing initial beliefs regardless of their accuracy. Third, authoritative sources such as professional medical organizations are rarely the most visible content on social media.

The protective effect of the physician as primary source (aOR = 0.51 for discordance) suggests that professional guidance is not merely informative but also attitudinally corrective. A physician who explains why an antibiotic is not needed, who describes the expected course of a viral illness, and who offers symptomatic management options may change not only what the patient knows but also what the patient believes. Evidence from randomized controlled trials supports this. Brief educational messages delivered during consultations reduce antibiotic demand without reducing patient satisfaction, and delayed prescribing strategies are effective for respiratory infections [50].

The sensitivity analyses confirmed that the primary findings are not artifacts of arbitrary cutoff choices. Varying the adequate knowledge cutoff from 60% to 50% or 70% changed the size of the discordant subgroup (from 103 participants at the 50% cutoff to 64 participants at the 70% cutoff, compared to the primary finding of 87 at the 60% cutoff) but did not change the pattern of predictors. Replacing the median practice cutoff with the 25th percentile (practice score ≤ 45.8) created a stricter definition of non-compliance, reducing the discordant subgroup to 49 participants. Again, the direction and significance of predictors remained unchanged. These sensitivity checks are consistent with methodological recommendations for KAP research, which emphasize that results should be robust to reasonable variations in threshold definitions [51].

The results argue against the continued reliance on knowledge-based educational campaigns as standalone interventions. Students in this study already knew, at adequate levels, the facts that such campaigns would deliver. The discordant subgroup specifically requires different approaches. Behavioral economics offers promising tools. Default options that require active choice to receive an antibiotic (such as delayed prescribing) have shown effectiveness in primary care settings. Social norm messaging that corrects misperceptions about peer behavior could reduce the influence of perceived family pressure.

The four-class LCA typology (Table 11, Fig. 5) can directly guide intervention design. Class 1 (Informed Adherents, 27.1%) does not require corrective intervention but may benefit from reinforcement messaging to sustain appropriate behaviors in a social environment that normalizes antibiotic misuse. Class 2 (Knowledgeable Non-Compliers, 24.1%) requires attitude change interventions, not knowledge transfer. Motivational interviewing, cognitive reframing, and social norm feedback are promising approaches for this group. Class 3 (Indifferent, 31.0%) may be reachable through standard educational campaigns. Class 4 (System Reliant, 17.7%) requires interventions that build personal knowledge while reinforcing trust in physicians.

The finding that medical faculty enrollment did not protect against discordance (aOR = 0.87, non-significant, Table 9) has concerning implications for medical education. Medical students in clinical years had higher knowledge than preclinical students (78.1% vs. 70.5%), but their practice scores were not significantly different. Knowledge accumulation during medical training does not automatically translate into appropriate personal antibiotic practices. Medical curricula should explicitly address the psychological and social drivers of antibiotic use, not only the pharmacological and microbiological aspects.

From the perspective of practicing internists in the same health system, the findings of this study are not merely academic. The 54.8% of students who take antibiotics without a prescription represent a population that will eventually present to primary care clinics and emergency departments with infections that are harder to treat. In clinical practice, we have observed a steady increase in community-acquired urinary tract infections resistant to first-line oral agents such as trimethoprim-sulfamethoxazole and ciprofloxacin [4]. Patients often report multiple prior courses of antibiotics obtained from pharmacies or leftover from previous prescriptions [4]. The 51.5% of students who reported family pressure to take antibiotics is also familiar. In the consultation room, we frequently encounter patients who express disappointment or distrust when an antibiotic is not prescribed, citing that a family member recovered quickly after taking antibiotics for similar symptoms [13]. This social dynamic, quantified in this study, is a real barrier to appropriate prescribing that medical education rarely addresses. The 34.3% of students who obtained antibiotics directly from pharmacies without a prescription represents a structural failure of regulatory enforcement. Until this pathway is closed, educational interventions aimed at patients will have limited impact because the behavior remains easy and without consequence. The Knowledge-practice discordance documented in this study is not a failure of student intelligence. It is a failure of a system that makes inappropriate use easy and appropriate use inconvenient. Internists, as the clinicians who manage the consequences of resistance, have a vested interest in shifting the focus of stewardship from patient education alone to system-level changes that include pharmacy regulation, public awareness campaigns targeting social norms, and clinical training in communication skills for managing patient expectations.

Several strengths of this study warrant mention. The stratified random sampling across all 16 faculties with proportional gender representation enhances generalizability within the university setting. The response rate of 96.6% is high for student surveys, reducing non-response bias. The use of a questionnaire that underwent pilot testing for face validity and demonstrated acceptable internal consistency across all three domains (Cronbach’s alpha 0.74 to 0.81) supports the reliability of the findings. The integration of multiple analytical methods provides convergent evidence for the central conclusions. The sensitivity analyses confirm robustness to alternative cutoff definitions.

Several limitations must be acknowledged. The cross-sectional design precludes causal inference; observed associations between knowledge, attitudes, and practices should be interpreted as correlational rather than causal, as temporality cannot be established. The temporal ordering assumed in the mediation analysis is theoretically plausible but not empirically established. Cross-sectional mediation estimates can be inflated by measurement timing and unmeasured confounding. Practices were self-reported, raising the possibility of social desirability bias [24]. The 12-month recall period for self-reported antibiotic use may have introduced recall bias, particularly for infrequent antibiotic events that occurred earlier in the recall period. Studies using prospective diaries or prescription record linkage have found that recall accuracy declines substantially after 3 to 6 months. If recall bias were present, it would likely be non-differential with respect to knowledge status; however, this possibility should be considered when interpreting the association between prior antibiotic use and discordance (aOR = 2.87). Future studies could employ shorter recall periods (e.g., 3 months) or objective data sources such as pharmacy records to validate self-reported use patterns. The single university design limits generalizability to other Egyptian institutions and to non-university populations. The practice score median split is data-driven rather than clinically validated, which may reduce comparability with studies using different thresholds. As with any dichotomization of continuous data, the median split involves some degree of arbitrariness and may not reflect clinically meaningful distinctions between ‘appropriate’ and ‘inappropriate’ practice [51]. However, sensitivity analyses using the 25th percentile cutoff (a stricter definition of non-compliance) confirmed that the direction and significance of all predictors remained unchanged, indicating that our findings are robust to the choice of cutoff. The LCA item selection involved some subjectivity. The study did not directly measure perceived behavioral control from the Theory of Planned Behavior framework, leaving that construct unexplored. Test-retest reliability was not assessed in this study, representing a limitation of the instrument validation. While internal consistency and content validity were established, future research should evaluate the temporal stability of the questionnaire through test-retest reliability testing.

Longitudinal studies following students through their academic careers would clarify whether discordance patterns persist. Qualitative research could explore the female paradox and family pressure mechanisms. Intervention studies should test whether attitude change strategies outperform standard education, particularly for Class 2 students. Given the strong association between social media reliance and poor outcomes, public health authorities should consider partnering with social media platforms to promote evidence-based content. Replication of the LCA typology in other Egyptian universities would establish generalizability.

## Conclusion

This study quantifies the knowledge-practice discordance among Egyptian university students and identifies its drivers. Over one-third of knowledgeable students (39.4%) reported inappropriate practices, representing 24.1% of the total sample, a subgroup for whom factual knowledge alone is insufficient. Latent class analysis revealed four behavioral phenotypes: Informed Adherents (27.1%), Knowledgeable Non-Compliers (24.1%), Indifferent (31.0%), and System Reliant (17.7%). Mediation analysis showed that attitudes account for 71% of the association between knowledge and practices, confirming that knowledge influences behavior primarily through attitudinal pathways.

These findings have immediate implications for antimicrobial stewardship in Egypt. First, educational campaigns delivering only factual information will not reach the nearly 40% of knowledgeable students who continue to misuse antibiotics. Second, interventions must be class-specific: attitude-focused for Knowledgeable Non-Compliers, knowledge-building for System Reliant, and reinforcement messaging for Informed Adherents. Third, discordance was strongly associated with prior antibiotic experience (aOR = 2.87) and social media reliance (aOR = 2.23), identifying modifiable targets beyond individual education.

Future research should test attitude-change versus knowledge-transfer interventions in randomized designs and evaluate pharmacist-led stewardship programs. Until then, stewardship in Egypt should prioritize prescription enforcement, physician-led counseling, and countering social media misinformation, with particular attention to female students and those with prior antibiotic experience, who are at the highest risk for discordant behavior.

## Data Availability

All relevant data are included in this published article.
